# Virtual screening–based discovery of AI-2 quorum sensing inhibitors that interact with an allosteric hydrophobic site of LsrK and their functional evaluation

**DOI:** 10.3389/fchem.2023.1185224

**Published:** 2023-05-24

**Authors:** Qianqian Shi, Huiqi Wen, Yijie Xu, Xu Zhao, Jing Zhang, Ye Li, Qingbin Meng, Fang Yu, Junhai Xiao, Xingzhou Li

**Affiliations:** ^1^ School of Petrochemical Engineering, Liaoning Petrochemical University, Fushun, China; ^2^ National Engineering Research Center for the Emergency Strategic Drug, Beijing Institute of Pharmacology and Toxicology, Beijing, China; ^3^ State Key Laboratory of Pathogen and Biosecurity, Institute of Microbiology and Epidemiology, Academy of Military Medical Sciences, Beijing, China; ^4^ State Key Laboratory of Toxicology and Medical Countermeasures, Beijing Institute of Pharmacology and Toxicology, Beijing, China; ^5^ Department of Hepatology, Fifth Medical Center of Chinese PLA General Hospital, Beijing, China; ^6^ Qionglai Medical Center Hospital, Chengdu, China; ^7^ The No 968 Hospital of PLA, Jinzhou, China

**Keywords:** ATP competitive inhibitors, LsrK, antibacterial agents, quorum sensing, virtual screening, molecular dynamics

## Abstract

**Introduction:** Quorum sensing (QS) is a bacterial intracellular and intercellular communication system that regulates virulence factor production, biofilm formation, and antibiotic sensitivity. Quorum-sensing inhibitors (QSIs) are a novel class of antibiotics that can effectively combat antibiotic resistance. Autoinducer-2 (AI-2) is a universal signaling molecule that mediates inter- and intraspecies QS systems among different bacteria. Furthermore, LsrK plays an important role in regulating the activity and stability of the intracellular AI-2 signaling pathway. Thus, LsrK is considered an important target for the development of QSIs.

**Methods:** We designed a workflow integrating molecular dynamic (MD) simulations, virtual screening, LsrK inhibition assays, cell-based AI-2-mediated QS interference assays, and surface plasmon resonance (SPR)-based protein affinity assays to screen for potential LsrK kinase inhibitors.

**Results:** MD simulation results of the LsrK/ATP complex revealed hydrogen bonds and salt bridge formation among four key residues, namely, Lys 431, Tyr 341, Arg 319, and Arg 322, which are critical for the binding of ATP to LsrK. Furthermore, MD simulation results indicated that the ATP-binding site has an allosteric pocket that can become larger and be occupied by small molecule compounds. Based on these MD simulation results, a constraint of forming at least one hydrogen bond with Arg 319, Arg 322, Lys 431, or Tyr 341 residues was introduced when performing virtual screening using Glide’s virtual screening workflow (VSW). In the meantime, compounds with hydrophobic group likely to interact with the allosteric hydrophobic pocket are preferred when performing visual inspection. Seventy-four compounds were selected for the wet laboratory assays based on virtual screening and the absorption, distribution, metabolism, and excretion (ADME) properties of these compounds. LsrK inhibition assays revealed 12 compounds inhibiting LsrK by more than 60% at a 200 μM concentration; four of these (Y205-6768, D135-0149, 3284–1358, and N025-0038) had IC_50_ values below 50 μM and were confirmed as ATP-competitive inhibitors. Six of these 12 LsrK inhibitors exhibited high AI-2 QS inhibition, of which, Y205-6768 had the highest activity with IC_50_ = 11.28 ± 0.70 μM. The SPR assay verified that compounds Y205-6768 and N025-0038 specifically bound to LsrK. MD simulation analysis of the docking complexes of the four active compounds with LsrK further confirmed the importance of forming hydrogen bonds and salt bridges with key basic amino acid residues including Lys 431, Tyr 341, Arg 319, and Arg 322 and filling the allosteric hydrophobic pocket next to the purine-binding site of LsrK.

**Discussion:** Our study clarified for the first time that there is an allosteric site near the ATP-binding site of Lsrk and that it enriches the structure–activity relationship information of Lsrk inhibitors. The four identified compounds showed novel structures, low molecular weights, high activities, and novel LsrK binding modes, rendering them suitable for further optimization for effective AI-2 QSIs. Our work provides a valuable reference for the discovery of QSIs that do not inhibit bacterial growth, thereby avoiding the emergence of drug resistance.

## 1 Introduction

Quorum sensing (QS) is an intercellular and intracellular communication system that allows bacteria to regulate many bacterial phenotypes by sensing interspecific or intraspecific populations (Antunes et al., 2010; Eickhoff and Bassler, 2018; Papenfort and Bassler, 2016). QS is involved in regulating various pathological processes, such as the induction of many bacteria density-dependent responses, including the synchronous production and secretion of virulence factors (Zhu et al., 2002; J. Li et al., 2021; Bansal et al., 2008; Antunes et al., 2010), bioluminescence, biofilm formation (Gu et al., 2020; Sun et al., 2020), changes in motility (Laganenka et al, 2016), cellular differentiation, and modification of antibiotic susceptibility (Grillo-Puertas et al., 2012; Patzelt et al., 2013). QS is mediated by signaling molecules called autoinducers (AIs). There are three main types of AIs: acylated homoserine lactones (AHL) utilized by gGram-negative bacteria; autoinducer peptides (AIPs) utilized by Gram-positive bacteria; and AI-2 identified in both Gram-positive and Gram-negative bacteria (Papenfort and Bassler, 2016). Other AIs include the *Pseudomonas* quinolone signal (PQS) (Hodgkinson et al., 2016; Dandela et al., 2018), diffusible signal factor (DSF) (He et al., 2022; Barber et al., 1997; Ryan et al., 2015), and *γ*-butyrolactone (Hur, Jang, and Sim, 2021; Takano, 2006). AI-2 is a unique AI that facilitates inter- and intraspecies communication and is thus defined as a “common language” of microbes (Federle and Bassler, 2003; Pereira, Thompson, and Xavier, 2013). Inter- and intraspecies communication through AI-2 QS has been demonstrated to coordinate critical features such as coaggregation, biofilm formation, and virulence (Duan et al., 2003). For instance, the synthesis of AI-2 by *Enterococcus faecalis* enhances *Escherichia coli (E. coli)* aggregation, coaggregation, and biofilm development (Laganenka, Colin, and Sourjik, 2016; Laganenka and Sourjik, 2018). Patients with cystic fibrosis and other diseases are often co-infected with *Pseudomonas aeruginosa* and Al-2 producing bacterial species such as *Staphylococcus aureus, Klebsiella pneumoniae,* and *Streptococcus mitis* (Laganenka and Sourjik, 2018; H. Li et al., 2015; Hotterbeekx et al., 2017; H. Li et al., 2017).

**FIGURE 1:**
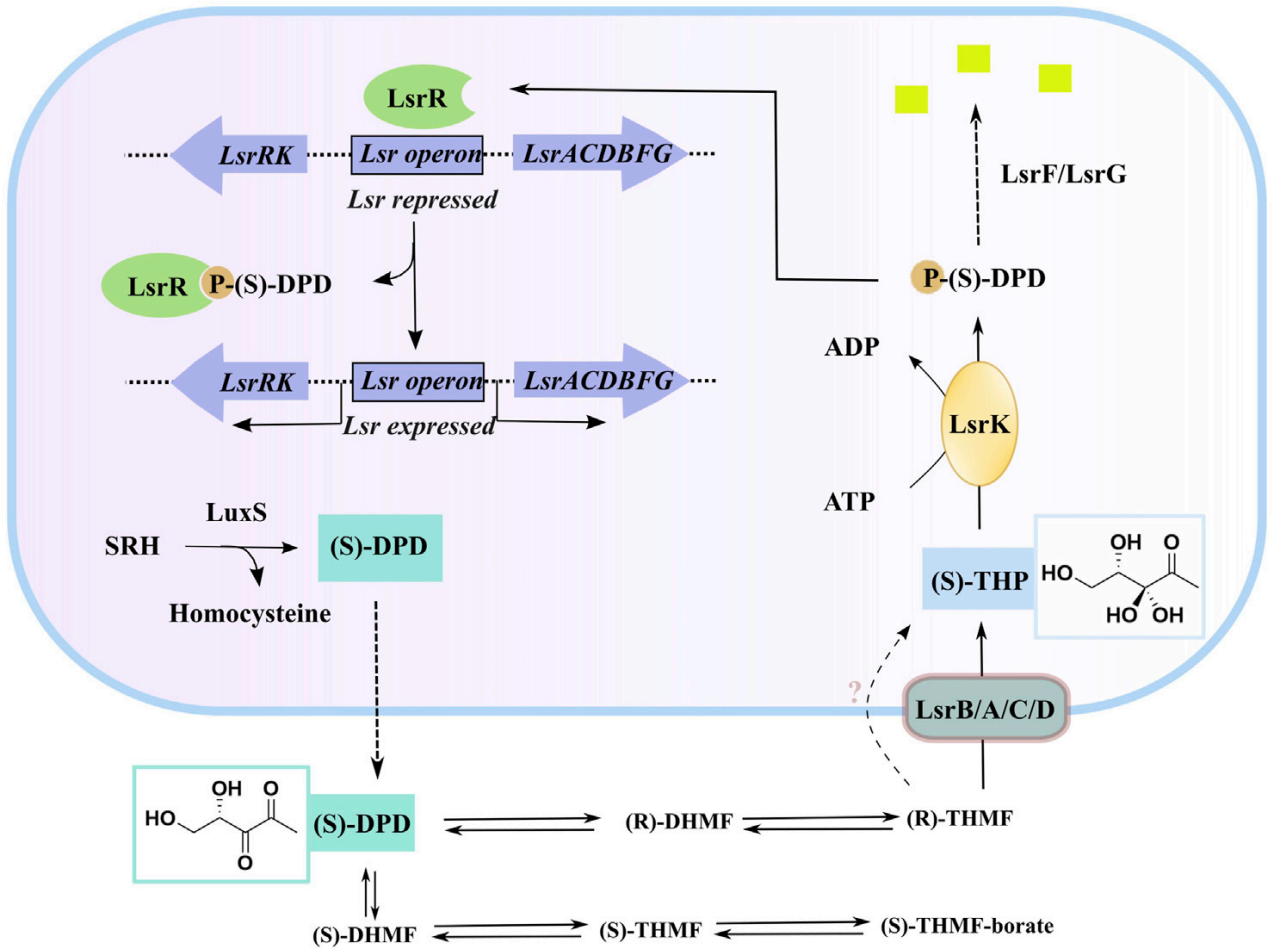
AI-2 mediated QS cascade—biosynthesis, transport, and degradation of AI-2 signal (Linciano et al., 2020). (“Equilibrium species of AI-2 in an aqueous environment” are shown in Supplementary Figure S1).

AI-2 is a set of (4*S*)-4,5-dihydroxy-2,3-pentanedione (*S*)-DPD derivatives that can rapidly convert to one another in solution (Stotani et al., 2019; Globisch et al., 2012). *S-ribosylhomocysteinase* (LuxS), which is found in more than 80 evolutionarily different bacterial species, may degrade *S-ribosylhomocysteine* (SRH) into adenine and (*S*)-DPD, the precursor of AI-2 (Lowery, Dickerson, and Janda, 2008; Zhu et al., 2013). Outside of the bacterial cytoplasmic membrane, linear (S)-DPD spontaneously rearranges into the cyclic isomers *S*-DHMF and *R*-DHMF. (Figure 1). Two cyclic tetrahydrated isomers, *S*-THMF and *R*-THMF, can be formed in the aqueous environment by hydration at C_3_ (Figure 1). X-ray crystallography revealed that *S*-THMF (Wang et al., 2018; Medarametla et al., 2021), in the form of S-THMF-borate, binds to the periplasmic protein LuxP (Chen et al., 2002), thereby activating QS in *Vibrio harveyi*. When the extracellular medium has an adequate amount of AI-2 in enteric bacteria, R-THFM is imported via the LsrBACD transporter, and its linear form (i.e., *S*-THP) is phosphorylated by LsrK, a member of the FGGY family of carbohydrate kinases (Hooshangi and Bentley, 2011) Finally, LsrG and LsrF process the resultant S-THP-phosphate [phospho-DPD (P-DPD, Figure 1)], to end the AI-2 signaling cycle (Ha et al., 2018).

P-DPD can bind to LsrR and undo the suppression of the LuxS-regulated (Lsr) promoter, thereby increasing the expression level of the transporter and positively regulating the AI-2-induced QS (Xavier and Bassler, 2005; Xavier et al., 2007). LsrK is the only known kinase that can phosphorylate DPD, and AI-2-induced QS has been reported to be markedly less intense in LsrK deletion–deficient strains than in wild-type strains (Zhu et al., 2013). Thus, using selective LsrK inhibitors to suppress QS could represent a valuable strategy for obtaining new antibiotics.

**FIGURE 2:**
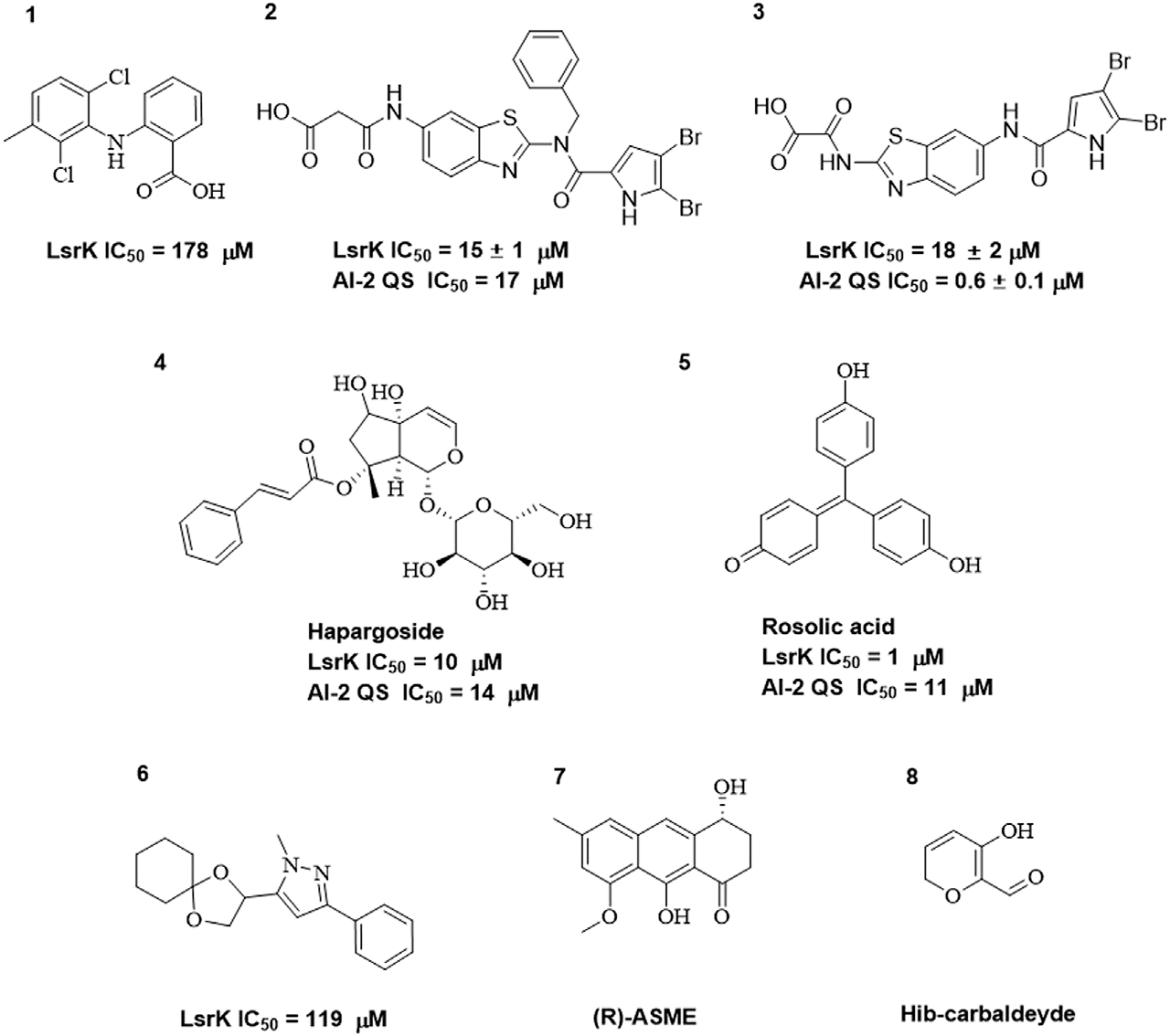
Chemical structure, LsrK inhibitory activity, and AI-2 QS inhibitory activity of the representative LsrK inhibitors.

As LsrK is a relatively new potential target, research on it is limited and mostly aided by computer-aided drug design. One type of LsrK inhibitor (such as compound 1, Figure 2) was identified through virtual screening using the LsrK homology model (Medarametla et al., 2018). The homology model demonstrates good structural agreement with the recently reported crystal structure of LsrK. Medarametla used molecular dynamics simulations to provide details of structural flexibility of these structures and variations in binding-site volumes, which indicates the LsrK binding site can accommodate ligands of different sizes. This study contributed to structure-based drug design targeting LsrK and provides a foundation for further research on phosphorylation of the active site of LsrK.

A cell-based assay for identifying AI-2-mediated QSIs has been developed, and several hits (such as compounds 2 and 3, Figure 2) were discovered as ATP-competitive LsrK inhibitors (Gatta et al., 2020). Natural products (such as compound 4, harpagoside, compound 5, and rosolic acid, Figure 2) identified via high-throughput screening (Gatta et al., 2019), and compound 6 (Figure 2), designed using a structure-based approach (Stotani et al., 2019), were identified to have LsrK and AI-2 QS inhibitory activities. Recently, Listro et al. used tryptophan fluorescence spectroscopy as a simple and reliable analytical method to identify promising LsrK inhibitor candidates [such as compound 7, (*R*)-ASME, ((*R*)-(−)-aloesaponol III 8-methyl ether), and 8, Hib-carbaldehyde, Figure 2], which can suppress biofilm production (Listro et al., 2023).

Nevertheless, these reported ATP-competitive LsrK inhibitors have limitations such as poor activity, low ligand efficiency, and ambiguous structure–activity relationships (SARs). Therefore, it is necessary to find new active and druggable LsrK inhibitors to combat antibiotic resistance. In this study, we first identified key interactions between LsrK and ATP using MD simulations then performed *in silico* virtual screening of large-scale commercial compound libraries using a glide-based docking approach with constrained search criteria. Next, wet laboratory assays identified four compounds with novel structures and explicit binding modes to LsrK, which were suitable for optimization as leads for effective AI-2 QSIs.

## 2 Materials and methods

All chemicals were purchased from Sigma-Aldrich (St. Louis, MO, United States) unless otherwise stated. DPD was purchased from the SHANGHAI ZZBIO Co., LTD. (Shanghai, China). The Kinase-Glo Max Luminescent Kinase Assay Kit was purchased from Promega (Madison, WI, United States). NTA sensor chips were purchased from GE Healthcare (Chicago, IL, United States). All compounds used as potential inhibitors of LsrK were purchased from Topscience Biotechnology Co. Ltd. (Shanghai, China). The QS reporter strain WHQ02 (*E. coli* BL21 ΔTolC pWHQ01) was donated by Huiqi Wen (Institute of Microbiology and Epidemiology, Academy of Military Sciences, Beijing, China). The plasmid pWHQ01 was constructed by cloning the *lsr* promoter and the *lux*CDABE luminescent gene. *Bam*HI and *Xho*I were purchased from New England Biolabs (Ipswich, MA, United States). pET-28a was purchased from Novagen (Madison, WI, United States). *Escherichia coli* BL21 (DE3) cells were purchased from TransGen Biotech Co., LTD. (Beijing, China). A Ni-NTA column was purchased from Sangon Biotech (Shanghai, China). Sodium dodecyl sulfate-polyacrylamide gel electrophoresis (SDS-PAGE) and the Bradford Protein Assay Kit were purchased from Thermo Fisher Scientific (Waltham, MA, United States).

### 2.1 Molecular docking, molecular dynamics, and binding free energy calculation

#### 2.1.1 Molecular docking

Three-dimensional conformations of the small molecules were generated using the LigPrep module (Schrödinger 2020.3). An OPLS3 force field was applied to produce low-energy conformers. Possible ionization states and tautomers were generated at pH 7.0 ± 2.0. The protein structure (PDB:5YA1) was prepared using Protein Preparation Wizard (Schrödinger 2020.3). Specifically, hydrogen atoms were added, the loop regions were refined, H-bond assignments were optimized, and restrained energy minimization (only the hydrogens) was then executed using the OPLS3 force field with default settings. Protein _chain_A (LsrK) and ATP were extracted from the protein complex for MD simulations. The receptor grid was generated using the Receptor Grid Generation module (Schrödinger 2020.3). The center of ATP (28.06(X), 26.37(Y), and 13.84(Z)) was designated as the grid center, and the grid box size was set to 1.2′, 1.2′, and 1.2 nm for x, y, and z, respectively.

#### 2.1.2 Molecular dynamics simulations

All MD simulations were performed using GROMACS 2021.3 (Páll et al., 2020). The LsrK/ligand complexes were simulated using the AMBER ff99SB-ILDN force field (Lindorff-Larsen et al., 2010) for the protein and GAFF force field for the ligands. The programs Acpype (Sousa da Silva and Vranken, 2012) and Ambertools were used to generate the ligand topologies for GROMACS. The B3LYP-D3(BJ)/ma-TZVP level was used to derive the partial atomic charges of ligands using the restrained electrostatic potential (RESP) method using Multiwfn in the gas phase and the IEFPCM solvation model (water environment). The RESP2 (0.5) charges (Schauperl et al., 2020) used in the MD simulations were derived based on the RESP charges for both environments. The octahedron box dimensions for periodic boundary condition (while keeping a minimum distance from any atom to the boundary of the box at 1 nm) were calculated as 6.9 nm × 5.2 nm × 7.4 nm. The TIP4P water model was used to conduct MD simulations in explicit solvation condition. Sodium ions (Na^+^) were added to the system for neutralization. The steepest descent algorithm was used for energy minimization, and the maximum force (Fmax) was set to not exceed 1,000 kJ/mol/nm. A Berendsen thermostat (Berendsen et al., 1984) and Parrinello-Rahman barostat (Parrinello and Rahman, 1981) were used for the temperature and pressure coupling, respectively. The system was equilibrated at 300 K and 1 bar using two consecutive 1,000 ps simulations with canonical NVT ensembles and isobaric NPT ensembles, respectively. MD simulations were then conducted under stable temperature and pressure conditions, with a 2 fs time step, LINCS algorithm for constraint handling, and a 1 nm cutoff for long-range interactions. After simulation completion, the GROMACS package was used to calculate the root-mean-square deviation (RMSD) and root-mean-square fluctuation (RMSF) relative to the crystal structure. To ensure the reliability and reproducibility of our results, we performed the experiment with three independent replicates of MD simulations using the same parameters. Similar results were obtained for all the three replicates, indicating the robustness and stability of our simulations.

#### 2.1.3 Estimation and decomposition of binding free energy by gmx_MMPBSA

The molecular mechanics/Poisson-Boltzmann surface area (MM/PBSA) in the gmx_MMPBSA tools (Valdés-Tresanco et al., 2021) was used to determine the thermodynamic stability of ligands inside the binding sites of the targets and to inspect the contribution of each residue in the binding pocket. In total, 100,000 frames of the LsrK/ATP complex were produced after equilibrium, and 20,000 frames for 200–400 ns were selected to calculate the binding energy and free energy decomposition. A total of 10,000 frames of LsrK/Y205-6768, LsrK/N025-0038, LsrK/D135-0149, and LsrK/3284-1358 complexes were produced after equilibrium, and 5,000 frames for 50–100 ns from each complex were selected to calculate the binding free energy and free energy decomposition, respectively. Default parameters were applied for all calculations.

#### 2.1.4 MD trajectory clustering analysis and volume calculation of the hydrophobic allosteric pocket

The MD trajectories of the LsrK/Y205-6768, LsrK/N025-0038, LsrK/D135-0149, and LsrK/3284-1358 complexes from 50 to 100 ns were selected for clustering analysis. Clustering analysis was conducted using the Gromos algorithm based on the RMSD values of the ligands, and the cutoff value was set at 0.25. One cluster for each complex was generated and superimposed for visual inspection by PyMOL 2.5 (The PyMOL Molecular Graphics System, Version 2.5 Schrödinger, LLC.)

To better determine the volume changes in the allosteric hydrophobic pocket, the corresponding residue set was extracted with a radius of 1 nm, centered on the Glu 345 residue in the crystal structure, using PyMOL 2.5 software, and the volume of the pocket was calculated using SiteMap (Schrödinger 2020.3). For the conformation at 20 ns during MD simulation, the same residue set centered on the Glu 345 residue was extracted, and the volume of the pocket was calculated using SiteMap. The difference in volume between the two conformations provided a relative quantitative measure of the conformational changes in the hydrophobic pocket.

#### 2.1.5 Virtual screening

The receptor grid was generated as described in Section 1.1. Nearly three million molecules from commercial compound libraries (Chemdiv and Enamine) were processed using the LigPrep module (Schrödinger suite) to develop the new compound 3D structure database described in Section 1.1.

According to the MD simulation of the LsrK/ATP complex, restrictions were introduced in the virtual screening to form at least one hydrogen bond with Arg 319, Arg 322, Lys 431, or Tyr 341 residues. The 3D structure database of newly generated compound was subjected to Glide VSW module (Schrödinger Suite) processing to obtain the initial hits. Glide-VSW is a hierarchical multi-precision docking protocol involving three levels of increasing docking precision: high-throughput virtual screening (HTVS), standard precision (SP), and extra precision (XP). The compounds were limited with an output score of the first 10% in each stage of HTVS and SP to the next round of docking, and the molecules with an output score of the first 10% in the XP stage were selected. Based on the XP Gscore, the top 400 molecules were selected and classified into 100 clusters by volume overlap. Compounds with hydrophobic groups that are likely to interact near the allosteric hydrophobic pocket were preferentially selected for visual inspection. Considering the structural diversity and ADME properties, 74 compounds were selected from the visually selected compounds and purchased for bioassay evaluation.

#### 2.1.6 Molecular property prediction

The molecular properties of the four compounds were assessed using the Ligand-Based ADME of AMDE and Molecular Properties module (Schrödinger 2020.3). The values of MW, cLogP, and PSA were extracted as references for the lead-likeness of the compounds.

### 2.2 Biological assay

#### 2.2.1 LsrK overexpression and purification

To express LsrK with a 6 × His-tag at the C-terminus, the LsrK encoding gene of *Salmonella typhimurium* was amplified using primers carrying restriction endonuclease sites *Bam*HI and *Xho*I. The amplified product was cloned into the expression vector pET-28a with a C-terminal 6 × His-tag and transfected into *E. coli* BL21 (DE3).

The transformed cells were selected on LB agar plates containing 50 μg/mL kanamycin. IPTG (0.5 mM) was added to induce protein synthesis. Then, the recombinant isolates were cultured at 37°C until mid-log exponential phase was reached. After overnight culturing, the cells were pelleted at 4°C 13,000 × *g* for 10 min, then resuspended in Lysis Buffer (50 mM NaH_2_PO_4_, 300 mM NaCl, pH 8.0), and sonicated on ice for 10 cycles with 30 s pulse and 30 s pause, and passed the supernatant through a 0.22 µm filter.

Then, the filtrate was loaded onto a Ni-NTA column, and proteins were eluted with 5 volumes of imidazole-containing buffer (50 mM NaH_2_PO_4_, 300 mM NaCl, 250 mM imidazole, pH 8.0) by way of a step gradient to recuperate the purified LsrK. The latter was then placed in a lysis buffer and dialyzing it overnight at 4°C using a 55 kDa molecular-mass-cutoff membrane bag. The molecular weight of lsrK was identified by utilizing sodium dodecyl sulfate 10% polyacrylamide gel electrophoresis (SDS-PAGE), followed by staining it with Coomassie blue. Subsequently, the Bradford Protein Assay Kit was used to quantify LsrK.

#### 2.2.2 LsrK inhibition assay

This assay was performed as previously described (Gatta et al., 2019), and the conditions were optimized. The assay was carried out at a final concentration of 300 nM LsrK, 100 μM ATP, and 300 μM DPD in a reaction buffer (pH 7.4) containing 25 mM TEA, 800 μM MgCl_2_, and 0.1 mg/mL BSA. 74 purchased compounds were dissolved in dimethyl sulfoxide (20% v/v DMSO in reaction buffer) at a concentration of 2 mM. Briefly, 10 μL DPD, 10 μL LsrK, and 5 μL compound were added to a 96-well plate, and 25 μL ATP or 25 μL reaction buffer was added after 30 min incubation. The plate was incubated for 10 min at 37°C, then 50 μL of kit reagent was added, and the plate was incubated at 37°C for 15 min. The luminescence signal was measured using an Enspire 2300 microplate reader (PerkinElmer). Dose-response tests were carried out to verify the activity of hits chosen by primary screening. (200 μM–6.25 µM) (Medarametla et al., 2018). The inhibition percentage of each tested compound was determined as previously described and IC_50_ was calculated using four logistic parameters.

#### 2.2.3 Glycerol kinase inhibition assay

The nine compounds that had an IC_50_ < 100 µM were dissolved in DMSO at a final concentration of 100 µM. The assay was carried out as previously described (Medarametla et al., 2018) for LsrK using a reaction mixture including 0.3 U/mL glycerol kinase from *E. coli*, 100 µM ATP, and 300 µM glycerol.

#### 2.2.4 SPR assay

His-tagged LsrK was immobilized in the NTA sensor chip (GE Healthcare) in Biacore 8 K using a running buffer consisting of 1 × PBS-T and 5% v/v DMSO. Serially diluted small molecules (concentration range: 50 to 1.56 µM) were injected. Intermediate concentration (12.5 µM) was set as a repeat, and 1 × PBS-T with 5% v/v DMSO was set as the control. The association time was set to 120 s; the dissociation time was set to 130 s; and the flow rate was set to 30 μL/min. The resulting data were fitted to the affinity binding model using Biacore Evaluation Software (GE Healthcare).

#### 2.2.5 Cell-based AI-2-mediated QS interference assay

WHQ02 cultured overnight were diluted 1:100 in fresh LB supplemented with 100 μg/mL ampicillin and grown at 30°C and until the logarithmic phase (OD_600_ = 0.4–0.6). The bacterial culture was centrifuged at 4,000 × *g* for 5 min, and the pellet was resuspended in fresh LB medium to prepare a suspension of 2 McFarland standard and added to the 96-well plate. In the experimental group, 74 compounds (final concentrations ranging from 100 to 3.125 μM) were added to reach a total volume of 100 µL. In addition, 100 µL LB medium with an equal volume of DMSO (negative control) or 100 µL LB supplemented with 2% glucose and the same volume of DMSO (positive control) were added. Bacteria was cultured at 37°C with shaking for 3 h. The fluorescence intensity of WHQ02 is directly regulated by the *lsr* promoter, reflecting the strength of QS. The luminescence and OD_600_ were measured using an Enspire 2300 microplate reader (Perkin Elmer). To further study the effect of compounds on bacterial growth, overnight cultured WHQ02 was diluted 1:1,000 with LB medium and added to 96 well plate. Then, the compounds were added to a 96-well plate (final concentration: 100 µM) and diluted in a gradient. The same volume of DMSO was added to the control group. The plates were incubated at 37°C for 18 h. OD_600_ values were recorded using an Enspire 2300 microplate reader. The experiment was performed in triplicate.

## 3 Results and discussion

### 3.1 Analysis of LsrK/ATP complex and virtual screening

#### 3.1.1 Analysis of LsrK/ATP dynamic interactions by MD simulation

**FIGURE 3:**
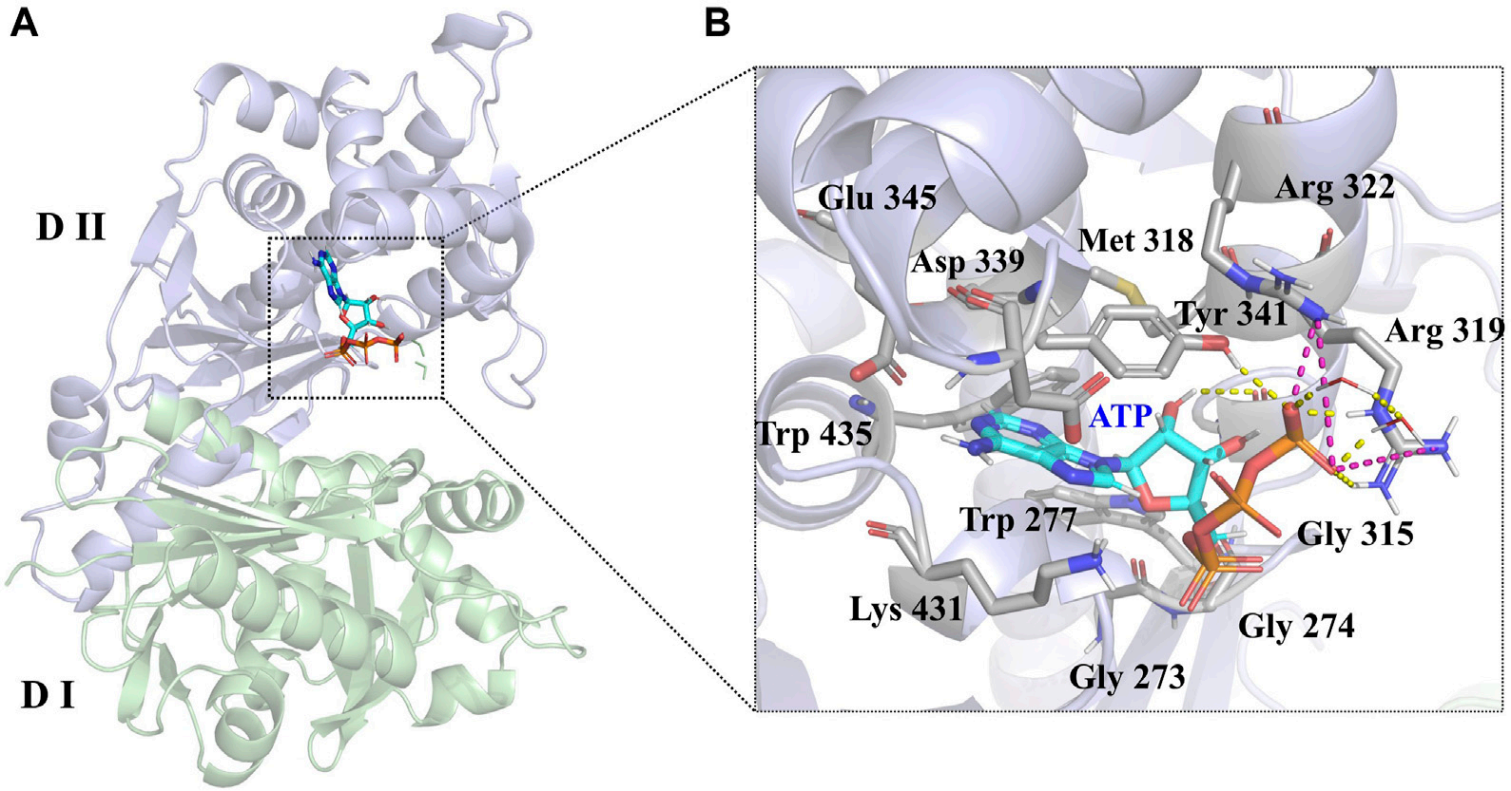
**(A)** Crystal structure of the LsrK/ATP complex (PDB: 5YA1). The two subunits of LsrK are shown as green cartoon (Domain Ⅰ) and purple cartoon (Domain Ⅱ). **(B)** A close-up of the interactions between ATP and LsrK (the involved residues are shown in gray stick). Key amino acid residues are represented in stick mode. Yellow dashed lines represent the hydrogen bonds, while purple dashed lines represent the salt bridges. Heteroatoms are color-coded (oxygen atoms in red, nitrogen atoms in dark blue, and phosphorus atoms in yellow).

LsrK consists of two domains (Figure 3A): an N-terminal domain (Domain I) and a C-terminal domain (Domain II). The ATP binding site consists of polar residues, such as Lys 341, Arg 319, and Arg 322, and a hydrophobic pocket composed of Tyr 341, Met 318, Trp 435, and Phe 394 residues. In the crystal structure (PDB:5YA1), the phosphate group of ATPs is near the solvent region and forms hydrogen bonds with Arg 319, Arg 322, Tyr 431, and the two crystal water molecules. The phosphate group also forms salt bridges with Arg 319 and Arg 322 residues. The 4-hydroxyl group of ribose forms a hydrogen bond with the Gly 315 residue (Figure 3B). Unlike other kinases, the purine of ATP did not form hydrogen bonds with LsrK in this crystal structure, piquing our interest and prompting us to use an MD simulation approach to analyze the binding mode of LsrK to ATP and to investigate the key amino acid residues.

**FIGURE 4:**
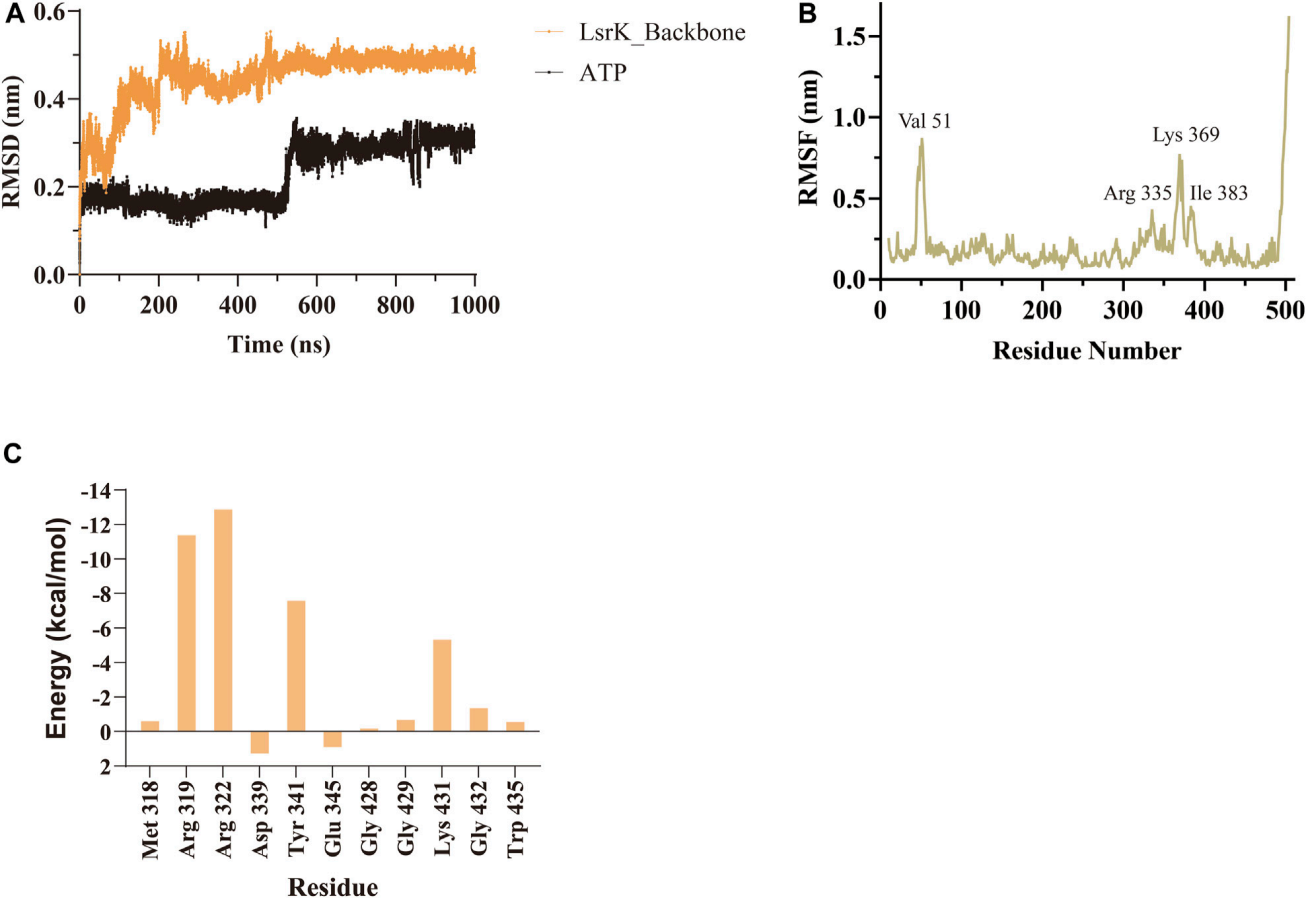
MD simulation results of LsrK/ATP complex. **(A)** RMSD values for LsrK backbone and ATP during 1000 ns MD simulations. **(B)** RMSF for the LsrK backbone residues during the 1000 ns simulation. **(C)** Calculated binding free energy and energy components of LsrK/ATP complex during 200–400 ns. ΔE_vdw_: contribution of the van der Waals energy; ΔE_ele_: contribution of the electrostatic energy; ΔE_PB_: contribution of the polar solvation energies; ΔE_nonPB_: contribution of the nonpolar solvation energies; ΔG_gas_: contribution of △E_vdw_ + ΔE_ele_; ΔG_slov_: contribution of ΔE_PB_ + ΔE_nonPB_; ΔG_total_: the final estimated binding free energy of ΔG_gas_ + ΔG_slov_.

A 1,000 ns MD simulation of the LsrK/ATP complex was performed using GROMACS 2021.3. The trajectory data of the production simulations were used to calculate the RMSD and RMSF. The RMSD values of proteins and ligands are important parameters for assessing the stability of the interactions between proteins and ligands during MD simulations. The RMSD values of the LsrK backbone demonstrated that LsrK underwent large conformational changes during the first 200 ns of MD simulation, while the conformations were relatively stable during the last 800 ns of MD simulation (Figure 4A). The RMSD values of ATP over time revealed drastic conformational changes at approximately 550 ns in the MD simulation (Figure 4A). The stability of each amino acid residue during the simulation was observed from the RMSF values. The RMSF plot showed that the conformational variability of residues in the ATP binding site, mainly located within the range of residue numbers 300–400, was significant (Figure 4B). The results indicated that the LsrK/ATP complex was not very stable during the simulation. In particular, the conformation of ATP and the amino acid residues of the ATP binding site underwent significant changes, and there were two different binding modes between ATP and LsrK according to the drastic change in ATP RMSD values during MD simulations.

**FIGURE 5:**
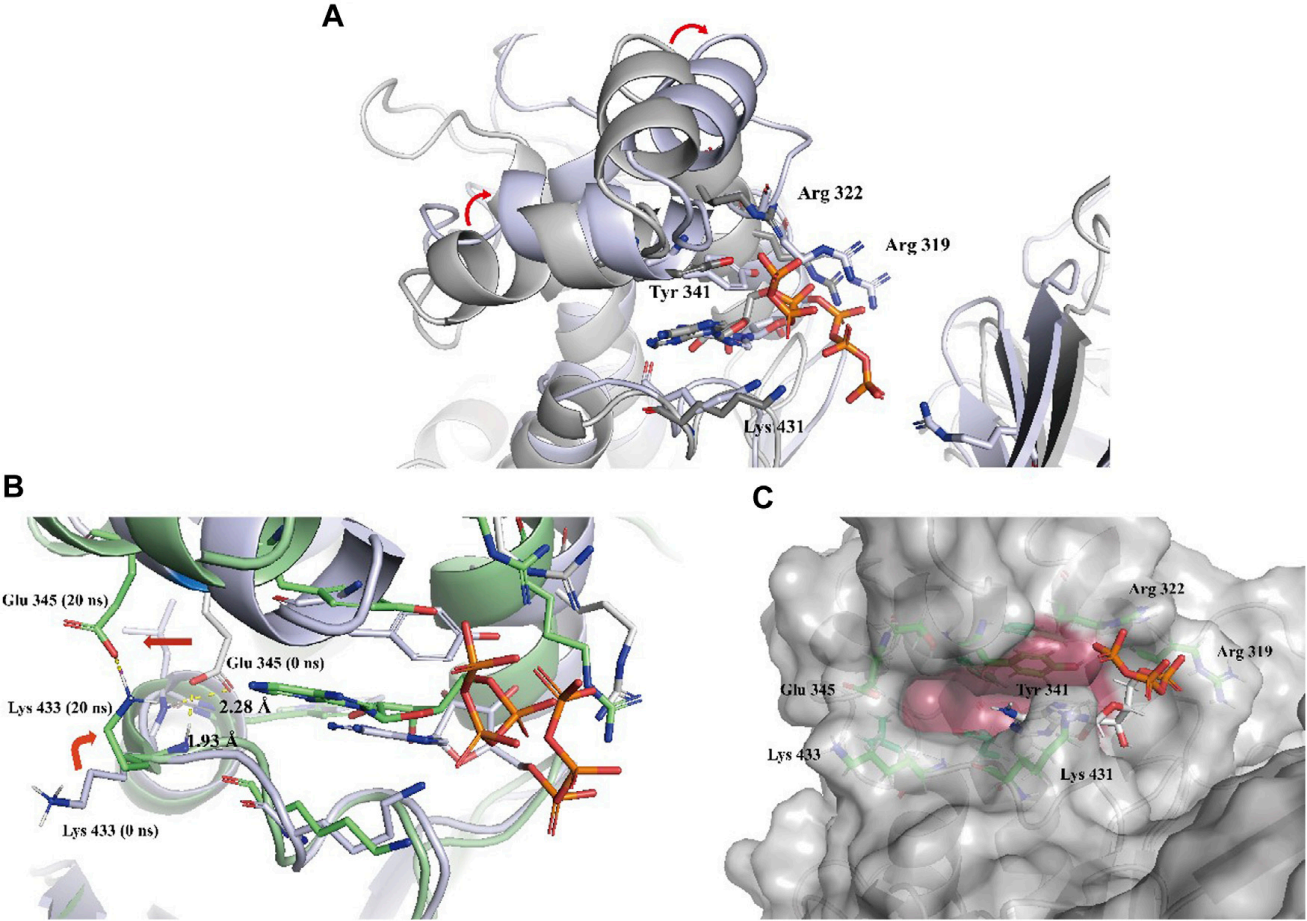
**(A)** Two representative conformations at 0 ns (the cartoon and C atoms of LsrK are in gray) and 850 ns (the cartoon and C atoms of LsrK are in purple) were selected to demonstrate the obvious conformational changes of the ATP binding site. **(B)** Conformational changes of Glu 345 and Lys 433 residues at 0 ns (the cartoon and C atoms of LsrK are in purple) and 20 ns (the cartoon and C atoms of LsrK are shown in green). **(C)**. Surface model of LsrK at 20 ns in the MD simulation. The surface of allosteric hydrophobic pocket residues is depicted in dark red.

To gain a more comprehensive understanding of the conformational change process, we extracted a total of 201 averaged structures from MD simulated trajectories at 5 ns intervals and verified them by visual inspection. The process of the ATP phosphate group extending to the binding pocket of the assumed substrate (DPD) was completed by a clockwise rotation of the entire C-terminal lobe and the continuous conformational changes of the Tyr 341 residue and three key basic residues, Lys 431, Arg 319, and Arg 322 (Figure 5A). The conformational fluctuation of ATP was mainly caused by the phosphate group extending to the presumed substrate (DPD) binding site. Interestingly, a conformational change in the hydrophobic pocket near the purine-binding site was observed during the MD simulation, which was significantly different from the crystal structure (Figure 5B). In the crystal structure, the side chain of the Glu 345 residue formed two hydrogen bonds with the main chain amide groups of Leu 434 and Trp 435 residues, with distances of 0.23 nm and 0.19 nm, respectively. Multiple MD simulations consistently showed that the deflection of the Glu 345 residue led to an increase in the volume of the hydrophobic pocket. Compared with the volume of the hydrophobic pocket in the crystal structure (0.119 nm^3^), that of the hydrophobic pocket after the conformational change increased by approximately 0.5-fold (0.179 nm^3^, Figure 5C). We believe that this potential conformational hydrophobic pocket is important for inhibitor design and could provide additional interaction sites for inhibitors to increase their affinity for LsrK.

Binding free energy calculations were used to analyze the affinity between ATP and LsrK, and free energy decomposition was used to analyze the hot amino acid residues that participate in ATP binding and induce conformational transformation of the ATP binding site. The binding free energy and free energy decomposition of each residue were calculated by selecting the stable binding period between ATP and LsrK (200–400 ns). The calculated binding free energy (ΔG_total_) was −40.86 ± 8.54 kcal/mol. The free energy decomposition results showed that residues Arg 319, Arg 322, Tyr341, and Lys431 made significant contributions to the binding free energy (Figure 4C).

In summary, the MD simulations provided us with two valuable pieces of information: (1) four amino acid residues, Tyr 341, Lys 431, Arg 319, and Arg 322, play an important role in maintaining the stable binding of ATP to LsrK, and (2) an allosteric hydrophobic pocket larger than that in the crystal structure can be generated near the ATP binding site of LsrK. This information is beneficial for the discovery and design of LsrK inhibitors.

#### 3.1.2 Structure-based virtual screening

Commercial compound libraries containing Chemdiv and Enamine, a total of approximately 3 million compounds, were processed and subjected to Glide-VSW module (Schrödinger suite) processing to obtain the initial hits. To improve the hit rate of virtual screening, a constraint search condition was added to form at least one hydrogen bond with the four key residues Arg319, Arg322, Tyr 341, and Lys 431. The presence of a potential allosteric hydrophobic pocket near the purine binding site is critical for the discovery of novel inhibitors, as it provides additional interaction sites for inhibitors for improving affinity. However, considering that the residue conformations of this allosteric site are very flexible, it would be risky to arbitrarily select a conformation obtained by MD simulation for virtual screening; therefore, we conservatively selected the LsrK conformation in the crystal structure (PDB) for virtual screening. However, in visual inspection, we focused on compounds with hydrophobic groups near the hydrophobic pocket to identify compounds that may interact with this site.

VSW performed screening with different precision in three stages: HTVS precision, SP, and extra precision (XP) screening. The top 10% of the compounds in each stage were selected for subsequent screening. Total 3,872 compounds passed extra precision screening, and the top 400 compounds with XP G-score value below −6.5 kcal/mol were divided into 100 clusters based on the volume overlap by ligand clustering module (Schrödinger 2020–3). Compounds with hydrophobic groups near the allosteric hydrophobic pocket were selected for visual inspection. Considering the hydrophobic interactions with the allosteric hydrophobic pocket and the binding diversity with LsrK and ADME properties, 74 commercially available compounds were shortlisted from the visually selected compounds and purchased for wet laboratory assays (Supplementary Table S1).

### 3.2 Biochemical assays

#### 3.2.1 LsrK inhibition assay

**FIGURE 6:**
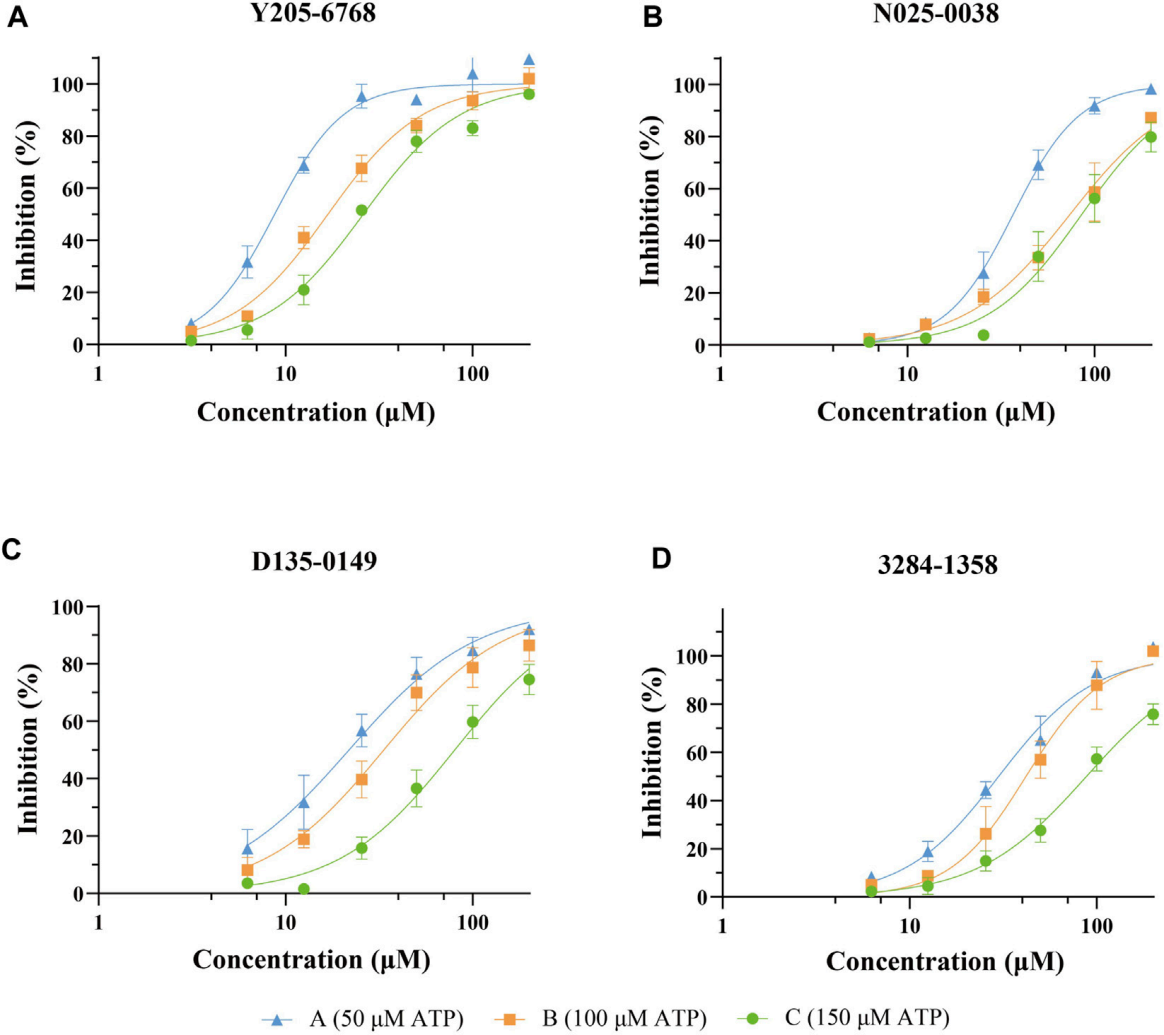
Dose-response curves against LsrK inhibition assay for compounds Y205-6768 **(A)**, N025-0038 **(B)**, D135-0149 **(C)**, and 3284–1358 **(D)** at different ATP (50, 100, and 150 μM). Data points are represented as the mean ± SD of three independent experiments (n = 3).

The 74 compounds were used in the LsrK inhibition assay at a final concentration of 200 µM with 100 µM ATP (Supplementary Table S1). ^43,44^ Twelve hits with inhibition levels above 60% were tested in a dose-response assay to determine their IC_50_ values (Table 1). Among these, four hits had IC_50_ values below 50 μM (Figure 6). Compound Y205-6768 had the highest inhibitory activity, with IC_50_ of 16.85 ± 0.76 μM, compounds 3284–1358, N025-0038, and D135-0149 also had good LsrK inhibition activities, with IC_50_ of 41.85 ± 2.57 μM, 43.70 ± 4.31 μM, and 33.46 ± 2.52 μM, respectively. The four hits with IC_50_ values below 50 μM were classified as ATP competitive inhibitors by measuring their IC_50_ values at different ATP concentrations (50, 100, and 150 μM). Considering that both glycerol kinase and LsrK belong to the FGGY family, glycerol kinase inhibition assays were performed to preliminarily verify the selectivity of the nine compounds with IC_50_ < 100 μM, and none showed obvious inhibition against glycerol kinase at 100 μM (results not shown).

**TABLE 1:** The IC_50_ values for the 12 positive hits tested against LsrK inhibition assay and cell-based AI-2 QS interference assay. Hits were tested at 200 μM with 100 μM ATP against LsrK inhibition assay. [A] Hits tested at 50 μM ATP against LsrK inhibition assay. [B] 100 μM ATP. [C] 150 μM ATP. [D] IC50 values for hits tested against cell-based QS interference assay. “N/A” means not applicable. IC50 values are represented as means ± SD of three independent experiments (n = 3).

Compound ID	Chemical structure	Inhibition (%)	IC50A (μM)	IC50B(μM)	IC50C(μM)	IC50D (μM)
Y205-6768	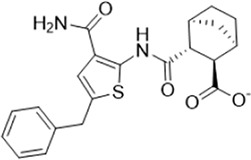	101.02 ± 8.39	8.73 ± 0.50	16.85 ± 0.76	26.05 ± 1.32	11.28 ± 0.70
3284–1358	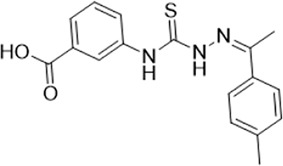	101.06 ± 3.82	29.89 ± 1.95	41.85 ± 2.57	88.09 ± 3.89	37.79 ± 3.18
Y204-6349	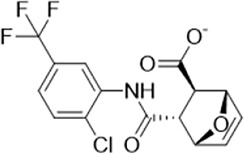	87.41 ± 2.60	N/A	75.14 ± 1.19	N/A	>100
D135-0149	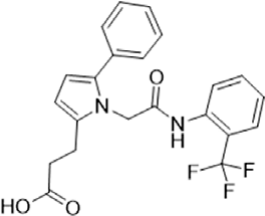	83.93 ± 6.33	21.80 ± 1.48	33.46 ± 2.52	80.10 ± 4.83	22.06 ± 1.66
N025-0038	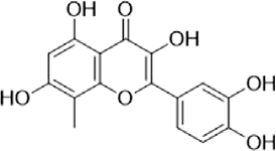	80.51 ± 2.31	26.64 ± 1.22	43.70 ± 4.31	67.98 ± 1.27	12.46 ± 0.99
3284–0335	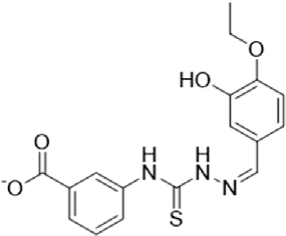	67.59 ± 4.88	N/A	84.86 ± 7.73	N/A	>100
2188–1861	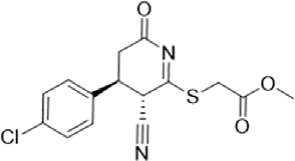	92.06 ± 6.68	N/A	128.76 ± 6.85	N/A	38.40 ± 3.35
5617–0915	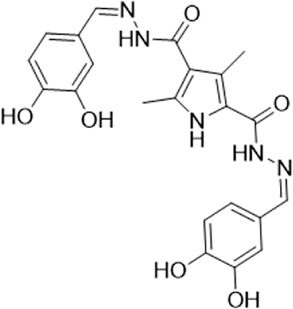	71.53 ± 4.33	N/A	79.42 ± 4.55	N/A	>100
Y040-9027	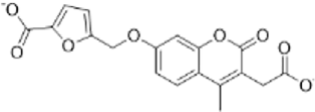	94.11 ± 6.72	N/A	90.04 ± 5.18	N/A	>100
3681–1274	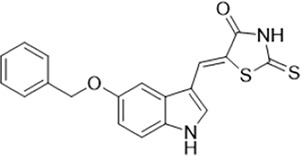	66.25 ± 3.54	N/A	133.20 ± 8.85	N/A	42.20 ± 2.15
4515–0182	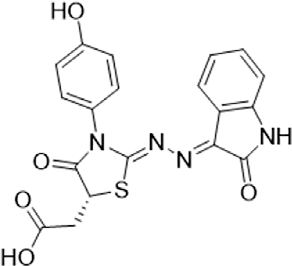	83.72 ± 9.63	N/A	52.06 ± 2.68	N/A	>100
Y502-2013	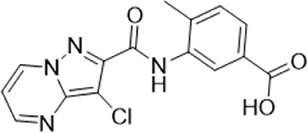	69.09 ± 8.64	N/A	139.91 ± 5.54	N/A	>100

#### 3.2.2 Cell-based AI-2-mediated QS interference assay

**FIGURE 7:**
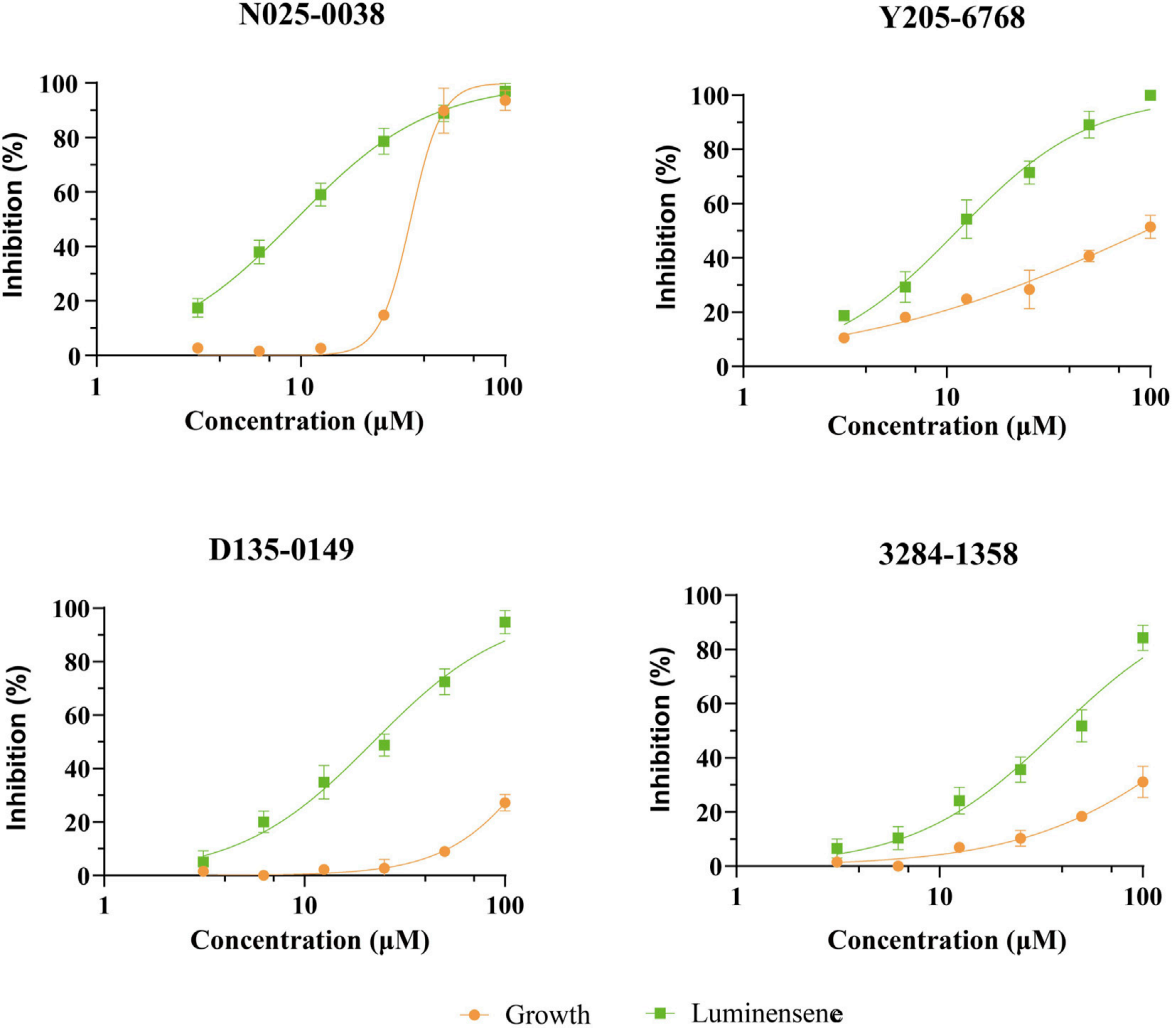
The QS inhibition and growth inhibition of N025-0038, Y205-6768, D135-0149, and 3681–1274 with concentrations ranging from 100 μM to 3.13 μM. The green and yellow curves represent the luminescence inhibition and the growth inhibition, respectively. Data points are represented as the mean ± SD of three independent experiments (n = 3).

The 12 hits showing >60% LsrK inhibition were tested for QS inhibition using cell-based AI-2-mediated QS interference assay, as previously described Baba et al., 2006). To ensure that lower luminescence was the result of QS inhibition not the chemical toxicity or antibacterial activity, strain growth inhibition was assessed concurrently. Since AI-2 QS mainly occurs at the end of the logarithmic bacterial growth and the bacteria count is stable, dosing at that time does not reflect the inhibitory effect of the compound on bacterial growth. Therefore, a parallel experiment to test the MIC of compounds at a low bacterial density was conducted, indirectly reflecting the potential growth inhibition to avoid false positives. Six compounds (Y205-6768, 3284–1358, D135–0149, N025-0038, 2188–1861, and 3681–1274), exhibited significant QS inhibitory activities (Figure 7 and Supplementary Figure S3), and three hits (Y205-6768, D135-0149, and 3284–1358) exhibited maximum growth inhibition (<40%) and maximum AI-2 QS inhibition (>80%) (Figure 7). Notably N025-0038 exhibited AI-2 QS inhibition at sub-minimum inhibitory concentrations (sub-MIC, Figure 7).

#### 3.2.3 SPR assay

**FIGURE 8:**
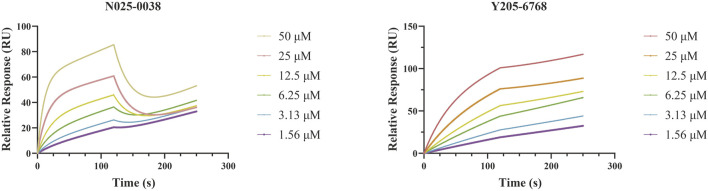
SPR Analysis of N025-0038 and Y205-6768. The graph depicts the binding curve (0–120 s) and dissociation curve (120–250 s) of the compounds used in concentrations ranging from 50 to 1.56 μM.

SPR assays were performed to further investigate the specific binding between active compounds and LsrK. Two compounds (Y205-6768 and N025-0038) with significant LsrK inhibitory effects were selected as the representative compounds for this assay. Considering that LsrK is easy to inactivate in the LsrK inhibition assay, the SPR assay was performed with the NTA chip rather than with the CM5 chip. The assay results demonstrated that Y205-6768 and N025-0038 exhibited LsrK specific binding, and the KD values were 8.49 × 10^−6^ M and 3.03 × 10^−5^ M, respectively (Figure 8). Although the KD values of Y205-6768 were close to those of N025-0038, the combination and dissociation curve of Y205-6768 had a more obvious trend of slow combination and dissociation compared to N025-0038.

### 3.3 Binding mode analysis and SAR exploration of hit compounds

Considering the flexibility of the ATP binding pocket and the existence of an allosteric hydrophobic pocket, molecular docking based on the LsrK/ATP crystal structure could not accurately predict the LsrK binding modes of these hits. Therefore, the MD simulation approach was used to determine the most likely structure of LsrK in complex with the four compounds (N025-0038, Y205-6768, D135-0149, and 3284–1358) with the highest QS inhibitory activities. Specifically, considering the docking results of these four compounds under XP precision in virtual screening as the initial conformation of MD simulation, 100 ns MD simulations was carried out for these four protein-ligand complexes. The representative conformations obtained by cluster analysis of 100 ns MD simulated trajectories were considered the most likely conformations of our LsrK-bound compounds.

**FIGURE 9:**
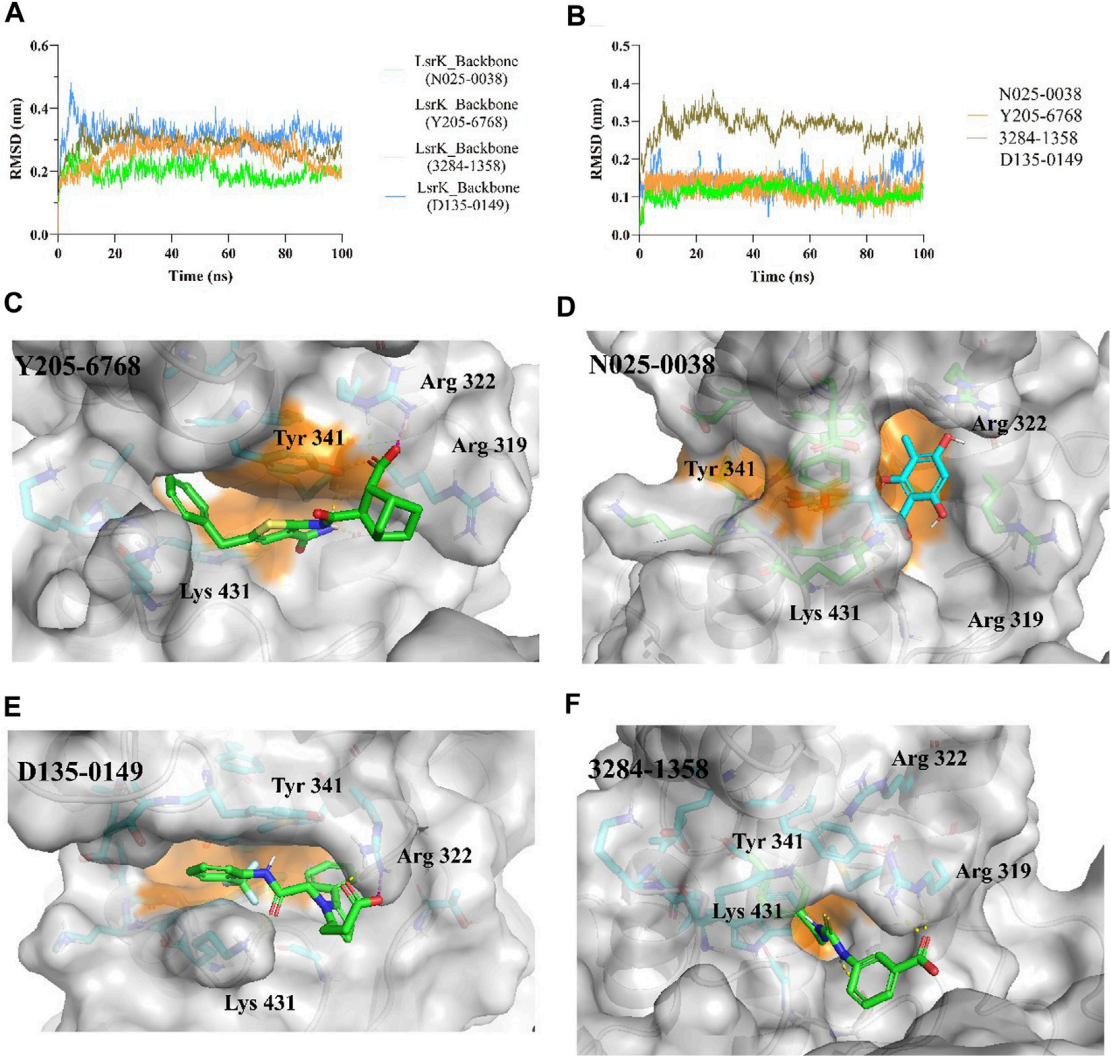
MD simulation results of LsrK/Y205-6768, LsrK/N025-0038, LsrK/D135-0149, and LsrK/3284–1358 complexes. **(A)** RMSD values *versus* time for LsrK backbone in four complexes during 100 ns MD simulations. **(B)** RMSD values *versus* time for N025-0038, Y205-6768, D135-0149, and 3284–1358 in four complexes during 100 ns MD simulations. **(C)** Representative conformation of LsrK/Y205-6768 complex. **(D)** Representative conformation of LsrK/N025-0038 complex. **(E)** Representative conformation of LsrK/D135-0149 complex. **(F)** Representative conformation of LsrK/3284–1358 complex. In the representative conformation, the key residues are depicted as sticks and C atoms are in blue. The surface of the residues in the allosteric hydrophobic pocket is depicted in orange.

The protein backbones did not fluctuate much during the 100 ns MD simulations, indicating that the protein, as a whole, was in equilibrium (Figure 9A). Furthermore, the conformations of N025-0038, Y205-6768, and D135-0149 were relatively stable, and the conformation of 3284–1358 fluctuated greatly (Figure 9B); however, there was no major change overall. The calculated binding free energies of Y205–6768, N025-0038, D135-0149, and 3284–1358 were −26.62 ± 8.64, −22.29 ± 5.64, −25.41 ± 7.07, and −18.29 ± 4.83 kcal/mol, respectively, which were consistent with the bioassay results. These results indicate that these four compounds (N025-0038, Y205-6768, D135-0149, and 3284–1358) can form stable complexes with LsrK.

According to the representative conformations of the four complexes in the MD simulation (Figures 9C–E, and F), all four compounds occupied the allosteric hydrophobic pocket via benzene rings, with or without substituent groups, which was not observed in the molecular docking results. Inhibitors (Gatta et al., 2020) with additional benzene rings exhibited higher LsrK inhibitory activities in the LsrK inhibition assay, which cannot be well explained by molecular docking. In the representative conformation, Y205-6768 formed hydrogen bonds or salt bridges with Gly 315, Tyr 341, and Arg 322. D135-0149 formed hydrogen bonds or salt bridges with Lys 431 and Arg 322; whereas 3284–1358 formed hydrogen bonds with Gly 315 and Arg 319. These findings confirm the importance of hydrogen bonds and salt bridges with key basic amino acid residues, such as Gly 315, Tyr 341, Arg 322, and Arg 319. This information is valuable for designing novel LsrK inhibitors in the future.

The rule of five (RO5; molecular weight <500 (MW), logP <5 (milog), hydrogen bond acceptors <10, hydrogen bond donors <5, and rotatable bonds) is a rule of thumb to evaluate drug likeness. When these rules are not violated, it means that the compound has better druggability. In this study, all four compounds were found to follow Lipinski’s rule of five (Table 2). Furthermore, these four compounds were found to possess novel structures, low molecular weights, favorable biological activities, and high affinities for LsrK, making them suitable lead compounds for the development of novel LsrK inhibitors.

**TABLE 2:** Chemical and physical properties of the 4 active compounds.

Compound ID	MW^a^	PSA^a^	cLogP	HBD^a^ count	HBA^a^ count	RB^a^ count	Lipinski #violations
Y205-6768	327.40	88.73	3.33	4	6	5	0
N025-0038	420.43	71.34	3.85	5	7	6	0
D135-0149	404.52	106.97	0.83	2	6	9	0
3284–1358	316.27	140.32	0.71	2	4	4	0

^a^ PSA, polar surface area; MW, molecular weight; HBA, H-bond acceptor; HBD, H-bond donor; RB, rotatable bond; cLogP, calculated LogP.

## 4 Conclusion

**FIGURE 10:**
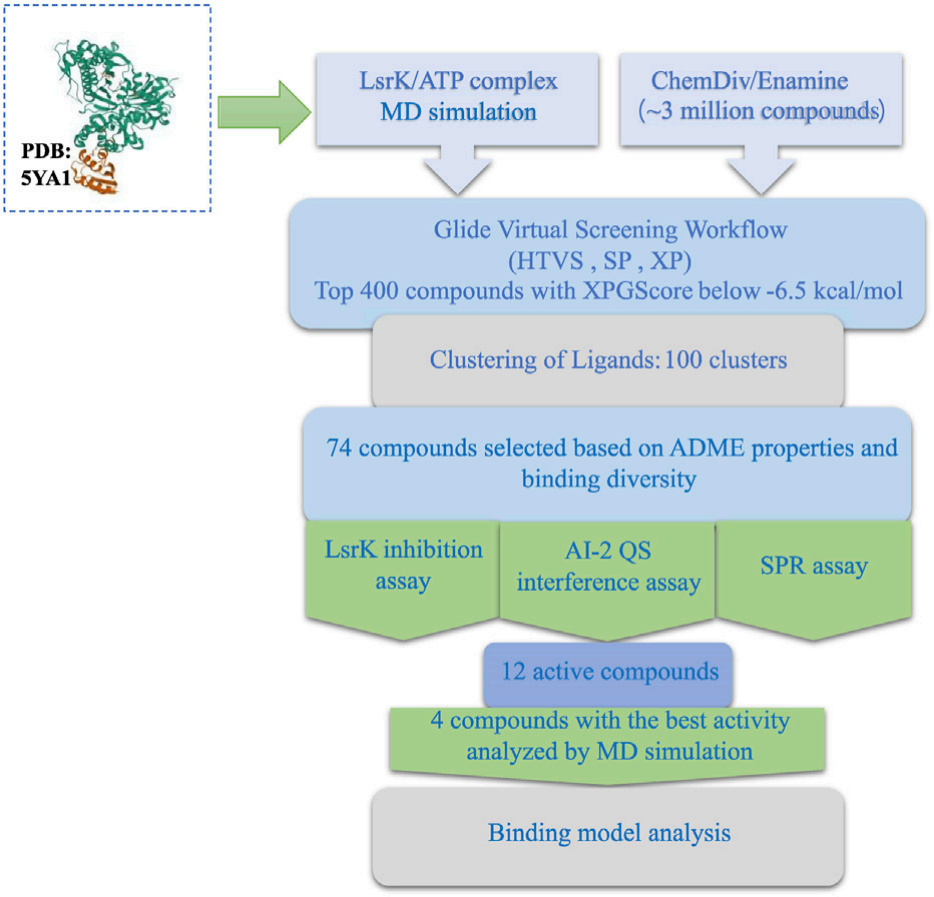
Stepwise flow diagram of the present work.

In this study, we developed a workflow for the virtual screening and bioassay-based evaluation of AI-2 QSIs against ATP binding site of LsrK. A stepwise flow diagram of the present work is illustrated in Figure 10.

We first analyzed the binding mode of ATP and LsrK by 1,000 ns MD simulations and obtained two valuable pieces of information: (1) Four amino acid residues (Arg 319, Arg 322, Tyr 341, and Lys 431) play an important role in maintaining a stable binding of ATP and LsrK; (2) Near the purine-binding site of LsrK, a significantly enlarged allosteric hydrophobic pocket, relative to that in the original crystal structure, was found, which can be occupied by small-molecule compounds.

In the following Glide-based virtual screening, we introduced the constraint of forming at least one hydrogen bond with these four key residues. Simultaneously, compounds with hydrophobic groups that are likely to interact near the allosteric hydrophobic pocket are preferred when performing a visual inspection. Considering the ligand efficiency, structural diversity, and ADME properties, 74 compounds were selected for wet laboratory assays. Twelve of the 74 purchased compounds inhibited the LsrK function, and six of these 12 compounds exhibited QS inhibition, with three of them (Y205-6768, D135-0149, and 3284–1358) exhibiting >80% QS inhibition% and <40% growth inhibition, while N025-0038 exhibited QS inhibition at sub-MIC concentrations. Our results confirmed that these AI-2 QSIs inhibited QS by specifically binding to the LsrK ATP-binding site and inhibiting LsrK phosphorylation. In addition, they have favorable characteristics such as a novel structure, low molecular weight, good biological activities, and druggability, and they are suitable as lead compounds for the development of new LsrK inhibitors, although partial compounds exhibited growth-inhibitory effects on bacteria. Binding mode analysis based on MD simulations confirmed the importance of the formation of hydrogen bonds and salt bridges with key basic amino acid residues and filling the allosteric hydrophobic pocket near the purine-binding site for LsrK inhibitors targeting the LsrK ATP-binding site. Our work provides a valuable reference for discovering QS inhibitors that are non-toxic and do not inhibit bacterial growth, thereby avoiding the emergence of drug resistance.

## Figures and Tables

**Table:** 

**QS**	quorum sensing
**QSIs**	quorum sensing inhibitors
**AIs**	Autoinducers
**AI-2**	autoinducer-2
**AIPs**	autoinducer peptides
**AI**	autoinducer
**MD**	molecular dynamics
**PQS**	*pseudomonas* quinolone signal
**DSF**	diffusible signal factor
**LuxS**	S-ribosylhomocysteinase
**R-DHMF**	(2R,4S)-2,4-dihydroxy-2-methyldihydrofuran-3-one
**R-THMF**	(2R,4S)-2-methyl-2,3,3,4-tetrahydroxy-tetrahydrofuran
**S-DHMF**	(2S,4S)-2,4-dihydroxy-2-methyldihydrofuran-3-one
**S-THMF**	(2S,4S)-2-methyl-2,3,3,4-tetrahydroxytetrahydrofuran
**S-THMF-borate**	(2S,4S)-2-methyl-2,3,3,4-tetrahydroxytetrahydrofuranborate
**BSA**	bovine serum albumin
**ATP**	adenosine triphosphate
**DPD**	3,4,4-trihydroxy-2-pentanone-5-phosphate
**S-THP**	S-3,3,4,5-tetrahydroxy-2-pentanone
**P-DPD**	S-3,3,4,5-tetrahydroxy-2-pentanone-5-phosphate
**S-DPD**	S-4,5-dihydroxy-2,3-pentanedione
**Lsr**	LuxS regulated
**MgCl**_**2**_	magnesium chloride
**μM**	micromolar
**AHL**	N-acyl homoserine lactones
**SRH**	S-ribosylhomocisteine
**PDB**	Protein Data Bank
**VSW**	virtual screening workflow
**HTVS**	high-throughput virtual screening
**SP**	standard precision
**XP**	extra-precision
**Fmax**	maximum force
**RMSD**	root-mean-square deviation
**RMSF**	root-mean-square fluctuation
**MM/PBSA**	Molecular mechanics/Poisson-Boltzmann surface area
**SPR**	surface plasmon resonance
**HTS**	high throughput screening
**SARs**	structure-activity relationships
**SDS-PAGE**	sodium dodecyl sulfate-polyacrylamide gel electrophoresis
**PSA**	polar surface area
**MW**	molecular weight
**HBA**	H-bond acceptor
**HBD**	H-bond donor
**RB**	Rotatable bond
**cLogP**	calculated LogP
**TEA**	Triethanolamine
**DMSO**	dimethyl sulfoxide
**NTA**	GE Healthcare
**OD**	optical density
**PBS-T**	phosphate buffer saline
**MIC**	minimum inhibitory concentration
**sub-MIC**	subminimum inhibitory concentration
**ADME**	Absorption, Distribution, Metabolism, Excretion.

## Data Availability

The original contributions presented in the study are included in the article/Supplementary Material, further inquiries can be directed to the corresponding authors.
