# Supplementary material for: Virtual screening–based discovery of AI-2 quorum sensing inhibitors that interact with an allosteric hydrophobic site of LsrK and their functional evaluation
Source: Front Chem. 2023 May 24;11:1185224. doi: 10.3389/fchem.2023.1185224 (PMC10244669; doi:10.3389/fchem.2023.1185224)
Supplement: Supplementary file 1 [file DataSheet1.docx]

Supplementary Material

Virtual Screening-based Discovery of AI-2 Quorum Sensing Inhibitors that Interact with an Allosteric Hydrophobic Site of LsrK and Their Functional Evaluation

Qianqian Shi^1,2†^, Wenqi Hui^3†^ and Yijie Xu^2,4†^, Xu Zhao^5^, Jing Zhang^6^, Ye Li^7^, Qingbin Meng^4^, Fang Yu^1,2*^, Junhai Xiao^2,4*^, Xingzhou Li^2,4*^

^1^School of Petrochemical Engineering, Liaoning Fushun 113001, China

^2^National Engineering Research Center for the Emergency Strategic Drug, Beijing Institute of Pharmacology and Toxicology, Beijing 100850, China

^3^State Key Laboratory of Pathogen and Biosecurity, Institute of Microbiology and Epidemiology, Academy of Military Medical Sciences, Beijing, 100071, China

^4^State Key Laboratory of Toxicology and Medical Countermeasures, Beijing Institute of Pharmacology and Toxicology, Beijing, 100850, China

^5^Department of Hepatology, Fifth Medical Center of Chinese PLA General Hospital, Beijing, 100039, China

^6^Qionglai Medical Center Hospital, Chengdu, 611530, China

^7^The No.968 Hospital of PLA, Jinzhou, 121000, China

†These authors contributed equally to this work and share first authorship

*** Correspondence:**

Fang Yu, fang.yu@lnpu.edu.cn
Xingzhou Li, lixz@bmi.ac.cn

Junhai Xiao, xiaojunhai@139.com

# Supplementary Figures and Tables

## Supplementary Figures


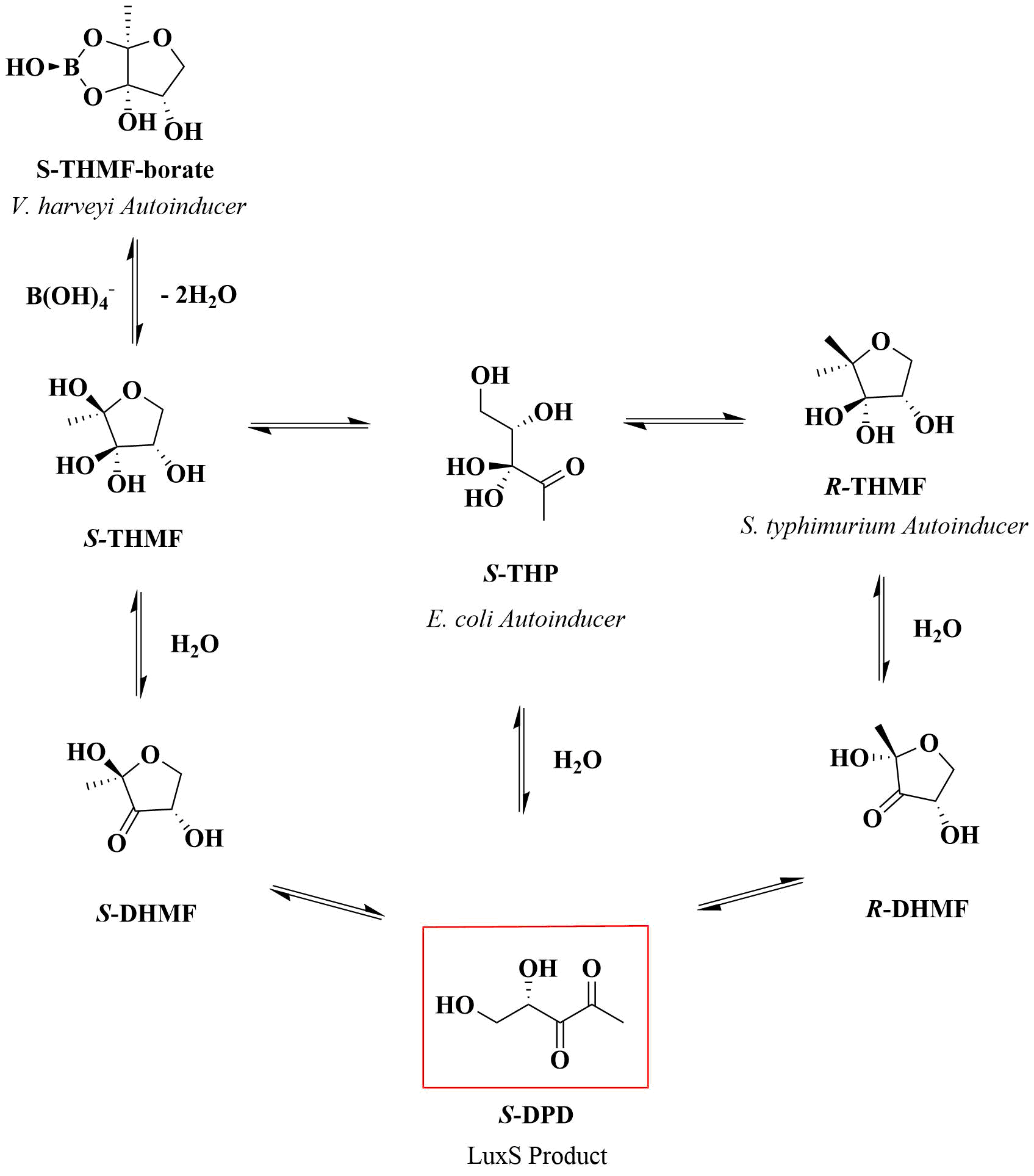


**Supplementary Figure 2.** Equilibrium species of AI- 2 in aqueous environment.

**
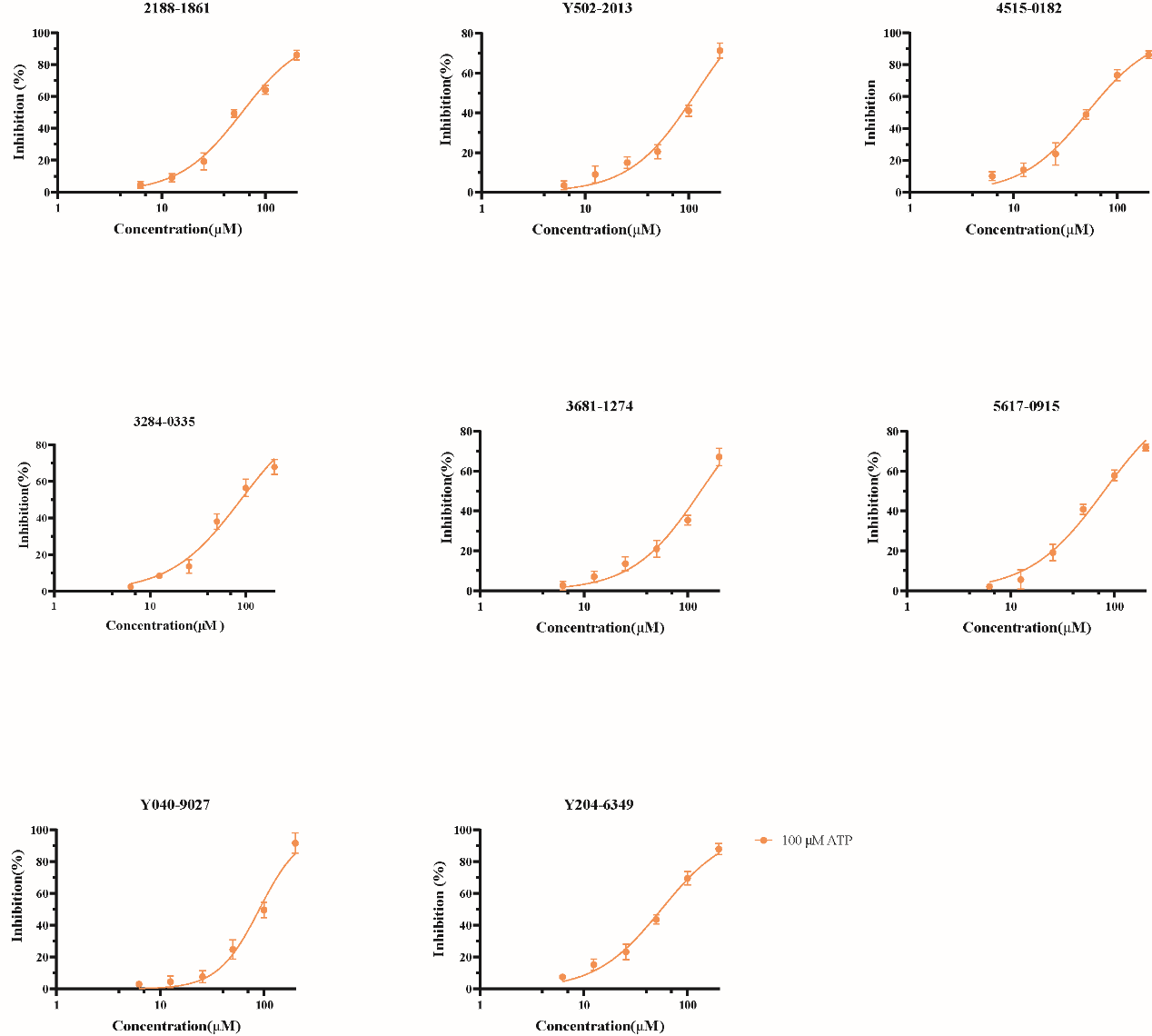
**

**Supplementary Figure 2.** LsrK inhibitors were tested for LsrK inhibition tested at 100 μM ATP. Data points represent the mean ± SD of three independent experiments (n = 3).


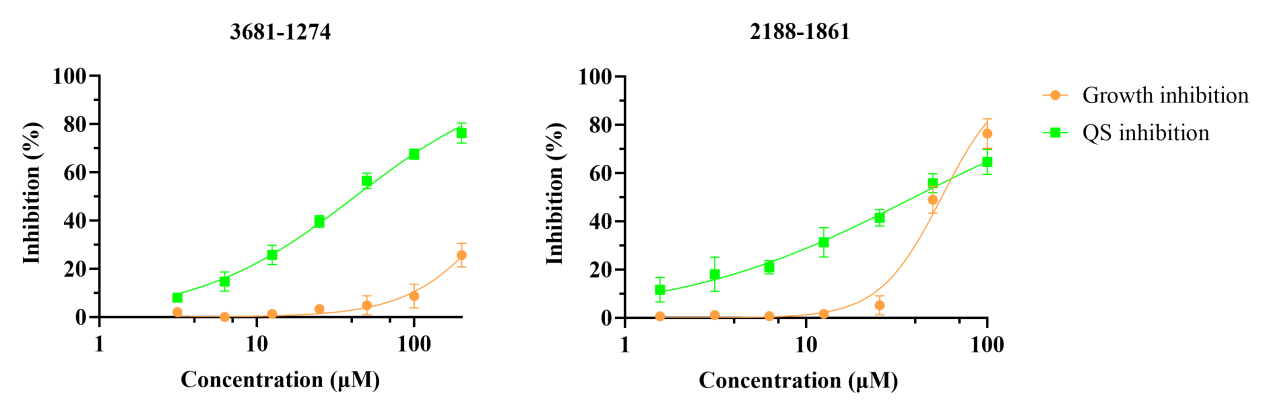


**Supplementary Figure 3.** The QS inhibition and growth inhibition of 3681-1274 and 2188-1861 with the concentration from 100 μM to 3.13 μM. Data points represent means ± SD from three independent experiments (n=3).

## Supplementary Tables

**Supplementary Table 1**. List of 74 compounds selected as potential LsrK inhibitors by virtual screening. Compounds were tested at 200 uM with 100 μM ATP against LsrK inhibition assay. “*” indicates that the color of the compound itself interferes with the determination of Luminescence values. inhibition (%) represented means ± SD from three independent experiments (n = 3).

| Compound ID | Structure | [Source](javascript:;) | Molecular Weight | XP GScore | Inhibition (%) |
| --- | --- | --- | --- | --- | --- |
| 3284 - 1358 | 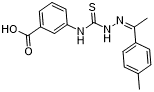 | [ChemDiv](http://www.baidu.com/link?url=lQnIdYT7ew-wpQhcPalb2rgUY_58qp3ks2kLNht-g4oqg-9on3FB4kFFhyWrOjiihP3EOTSOWklD8-nzNYMQLexE5qEYJdr4AE62epQ2OMq) | 327.4 | - 9.199 | 101.06 ± 3.82 |
| Y205 - 6768 | 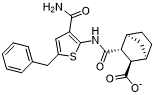 | ChemDiv | 398.476 | - 9.531 | 101.02 ± 8.39 |
| Y040 - 9027 | 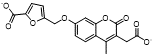 | ChemDiv | 358.304 | - 9.306 | 94.11 ± 6.72 |
| 2188 - 1861 | 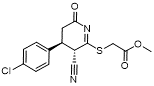 | [ChemDiv](http://www.baidu.com/link?url=lQnIdYT7ew-wpQhcPalb2rgUY_58qp3ks2kLNht-g4oqg-9on3FB4kFFhyWrOjiihP3EOTSOWklD8-nzNYMQLexE5qEYJdr4AE62epQ2OMq) | 336.792 | - 10.239 | 92.06 ± 9.68 |
| Y204 - 6349 | 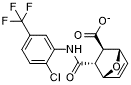 | [ChemDiv](http://www.baidu.com/link?url=lQnIdYT7ew-wpQhcPalb2rgUY_58qp3ks2kLNht-g4oqg-9on3FB4kFFhyWrOjiihP3EOTSOWklD8-nzNYMQLexE5qEYJdr4AE62epQ2OMq) | 361.704 | - 8.779 | 87.41 ± 2.60 |
| D135 - 0149 | 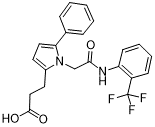 | [ChemDiv](http://www.baidu.com/link?url=lQnIdYT7ew-wpQhcPalb2rgUY_58qp3ks2kLNht-g4oqg-9on3FB4kFFhyWrOjiihP3EOTSOWklD8-nzNYMQLexE5qEYJdr4AE62epQ2OMq) | 416.399 | - 10.154 | 83.93 ± 6.33 |
| 4515 - 0182 | 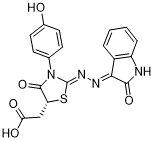 | [ChemDiv](http://www.baidu.com/link?url=lQnIdYT7ew-wpQhcPalb2rgUY_58qp3ks2kLNht-g4oqg-9on3FB4kFFhyWrOjiihP3EOTSOWklD8-nzNYMQLexE5qEYJdr4AE62epQ2OMq) | 410.403 | - 10.144 | 83.72 ± 9.63 |
| N025 - 0038 | 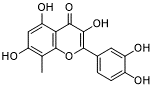 | [ChemDiv](http://www.baidu.com/link?url=lQnIdYT7ew-wpQhcPalb2rgUY_58qp3ks2kLNht-g4oqg-9on3FB4kFFhyWrOjiihP3EOTSOWklD8-nzNYMQLexE5qEYJdr4AE62epQ2OMq) | 316.267 | - 10.993 | 80.51 ± 2.31 |
| 5617 - 0915 | 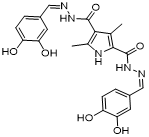 | [ChemDiv](http://www.baidu.com/link?url=lQnIdYT7ew-wpQhcPalb2rgUY_58qp3ks2kLNht-g4oqg-9on3FB4kFFhyWrOjiihP3EOTSOWklD8-nzNYMQLexE5qEYJdr4AE62epQ2OMq) | 451.438 | - 11.171 | 71.53 ± 4.33 |
| Y502 - 2013 | 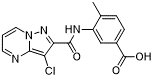 | [ChemDiv](http://www.baidu.com/link?url=lQnIdYT7ew-wpQhcPalb2rgUY_58qp3ks2kLNht-g4oqg-9on3FB4kFFhyWrOjiihP3EOTSOWklD8-nzNYMQLexE5qEYJdr4AE62epQ2OMq) | 330.73 | - 8.112 | 69.09 ± 8.64 |
| 3284 - 0335 | 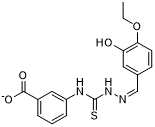 | [ChemDiv](http://www.baidu.com/link?url=lQnIdYT7ew-wpQhcPalb2rgUY_58qp3ks2kLNht-g4oqg-9on3FB4kFFhyWrOjiihP3EOTSOWklD8-nzNYMQLexE5qEYJdr4AE62epQ2OMq) | 359.399 | - 10.391 | 67.59 ± 4.88 |
| D665 - 0557 | 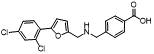 | [ChemDiv](http://www.baidu.com/link?url=lQnIdYT7ew-wpQhcPalb2rgUY_58qp3ks2kLNht-g4oqg-9on3FB4kFFhyWrOjiihP3EOTSOWklD8-nzNYMQLexE5qEYJdr4AE62epQ2OMq) | 376.238 | - 10.115 | 66.34 ± 10.57 |
| 3681 - 1274 | 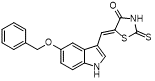 | [ChemDiv](http://www.baidu.com/link?url=lQnIdYT7ew-wpQhcPalb2rgUY_58qp3ks2kLNht-g4oqg-9on3FB4kFFhyWrOjiihP3EOTSOWklD8-nzNYMQLexE5qEYJdr4AE62epQ2OMq) | 366.452 | - 7.413 | 66.25 ± 3.54 |
| E470 - 0571 | 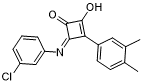 | [ChemDiv](http://www.baidu.com/link?url=lQnIdYT7ew-wpQhcPalb2rgUY_58qp3ks2kLNht-g4oqg-9on3FB4kFFhyWrOjiihP3EOTSOWklD8-nzNYMQLexE5qEYJdr4AE62epQ2OMq) | 311.767 | - 9.566 | 59.98 ± 7.93 |
| Y040 - 4090 | 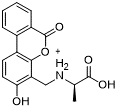 | [ChemDiv](http://www.baidu.com/link?url=lQnIdYT7ew-wpQhcPalb2rgUY_58qp3ks2kLNht-g4oqg-9on3FB4kFFhyWrOjiihP3EOTSOWklD8-nzNYMQLexE5qEYJdr4AE62epQ2OMq) | 313.309 | - 9.988 | 58.29 ± 6.97 |
| Z196022762 | 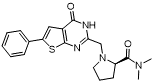 | [Enamine](http://www.baidu.com/link?url=lQnIdYT7ew-wpQhcPalb2rgUY_58qp3ks2kLNht-g4oqg-9on3FB4kFFhyWrOjiihP3EOTSOWklD8-nzNYMQLexE5qEYJdr4AE62epQ2OMq) | 382.479 | - 8.758 | 57.11 ± 2.22 |
| 2125 - 0471 | 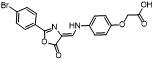 | [ChemDiv](http://www.baidu.com/link?url=lQnIdYT7ew-wpQhcPalb2rgUY_58qp3ks2kLNht-g4oqg-9on3FB4kFFhyWrOjiihP3EOTSOWklD8-nzNYMQLexE5qEYJdr4AE62epQ2OMq) | 417.215 | - 10.207 | 51.30 ± 5.08* |
| F293 - 0488 | 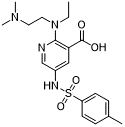 | [ChemDiv](http://www.baidu.com/link?url=lQnIdYT7ew-wpQhcPalb2rgUY_58qp3ks2kLNht-g4oqg-9on3FB4kFFhyWrOjiihP3EOTSOWklD8-nzNYMQLexE5qEYJdr4AE62epQ2OMq) | 406.499 | - 10.38 | 50.73±5.74* |
| Y041 - 7770 | 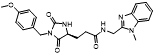 | [ChemDiv](http://www.baidu.com/link?url=lQnIdYT7ew-wpQhcPalb2rgUY_58qp3ks2kLNht-g4oqg-9on3FB4kFFhyWrOjiihP3EOTSOWklD8-nzNYMQLexE5qEYJdr4AE62epQ2OMq) | 435.482 | - 10.222 | 50.41 ± 5.23 |
| Y041 - 2370 | 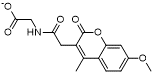 | [ChemDiv](http://www.baidu.com/link?url=lQnIdYT7ew-wpQhcPalb2rgUY_58qp3ks2kLNht-g4oqg-9on3FB4kFFhyWrOjiihP3EOTSOWklD8-nzNYMQLexE5qEYJdr4AE62epQ2OMq) | 362.338 | - 9.735 | 50.32 ± 9.15 |
| C708 - 0161 | 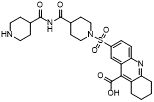 | [ChemDiv](http://www.baidu.com/link?url=lQnIdYT7ew-wpQhcPalb2rgUY_58qp3ks2kLNht-g4oqg-9on3FB4kFFhyWrOjiihP3EOTSOWklD8-nzNYMQLexE5qEYJdr4AE62epQ2OMq) | 528.622 | - 9.533 | 48.11 ± 8.96 |
| 5794 - 0692 | 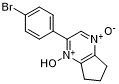 | [ChemDiv](http://www.baidu.com/link?url=lQnIdYT7ew-wpQhcPalb2rgUY_58qp3ks2kLNht-g4oqg-9on3FB4kFFhyWrOjiihP3EOTSOWklD8-nzNYMQLexE5qEYJdr4AE62epQ2OMq) | 307.146 | - 8.52 | 45.48 ± 2.18 |
| D135 - 0151 | 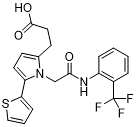 | [ChemDiv](http://www.baidu.com/link?url=lQnIdYT7ew-wpQhcPalb2rgUY_58qp3ks2kLNht-g4oqg-9on3FB4kFFhyWrOjiihP3EOTSOWklD8-nzNYMQLexE5qEYJdr4AE62epQ2OMq) | 422.421 | - 10.561 | 45.12 ± 7.41 |
| 8002 - 4370 | 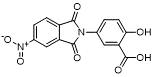 | [ChemDiv](http://www.baidu.com/link?url=lQnIdYT7ew-wpQhcPalb2rgUY_58qp3ks2kLNht-g4oqg-9on3FB4kFFhyWrOjiihP3EOTSOWklD8-nzNYMQLexE5qEYJdr4AE62epQ2OMq) | 328.237 | - 8.733 | 43.24 ± 3.92 |
| D715 - 2786 | 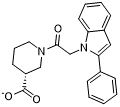 | [ChemDiv](http://www.baidu.com/link?url=lQnIdYT7ew-wpQhcPalb2rgUY_58qp3ks2kLNht-g4oqg-9on3FB4kFFhyWrOjiihP3EOTSOWklD8-nzNYMQLexE5qEYJdr4AE62epQ2OMq) | 362.427 | - 9.726 | 40.9 ± 4.15 |
| 8020 - 2738 | 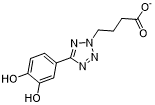 | [ChemDiv](http://www.baidu.com/link?url=lQnIdYT7ew-wpQhcPalb2rgUY_58qp3ks2kLNht-g4oqg-9on3FB4kFFhyWrOjiihP3EOTSOWklD8-nzNYMQLexE5qEYJdr4AE62epQ2OMq) | 264.24 | - 8.436 | 39.14 ± 1.81 |
| Y500 - 7045 | 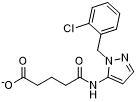 | [ChemDiv](http://www.baidu.com/link?url=lQnIdYT7ew-wpQhcPalb2rgUY_58qp3ks2kLNht-g4oqg-9on3FB4kFFhyWrOjiihP3EOTSOWklD8-nzNYMQLexE5qEYJdr4AE62epQ2OMq) | 321.763 | - 8.451 | 37.69 ± 9.30 |
| 8020 - 3618 | 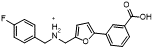 | [ChemDiv](http://www.baidu.com/link?url=lQnIdYT7ew-wpQhcPalb2rgUY_58qp3ks2kLNht-g4oqg-9on3FB4kFFhyWrOjiihP3EOTSOWklD8-nzNYMQLexE5qEYJdr4AE62epQ2OMq) | 325.339 | - 6.556 | 37.56± 3.79 |
| C276 - 0318 | 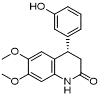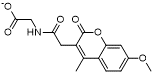 | [ChemDiv](http://www.baidu.com/link?url=lQnIdYT7ew-wpQhcPalb2rgUY_58qp3ks2kLNht-g4oqg-9on3FB4kFFhyWrOjiihP3EOTSOWklD8-nzNYMQLexE5qEYJdr4AE62epQ2OMq) | 299.326 | - 9.297 | 36.4 ± 3.94 |
| Y040 - 2672 |  | [ChemDiv](http://www.baidu.com/link?url=lQnIdYT7ew-wpQhcPalb2rgUY_58qp3ks2kLNht-g4oqg-9on3FB4kFFhyWrOjiihP3EOTSOWklD8-nzNYMQLexE5qEYJdr4AE62epQ2OMq) | 305.287 | - 8.899 | 34.58 ± 8.76 |
| 2188 - 0739 | 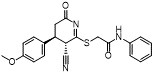 | [ChemDiv](http://www.baidu.com/link?url=lQnIdYT7ew-wpQhcPalb2rgUY_58qp3ks2kLNht-g4oqg-9on3FB4kFFhyWrOjiihP3EOTSOWklD8-nzNYMQLexE5qEYJdr4AE62epQ2OMq) | 393.459 | - 6.615 | 34.11 ± 6.23 |
| 3698 - 0088 | 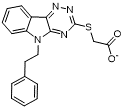 | [ChemDiv](http://www.baidu.com/link?url=lQnIdYT7ew-wpQhcPalb2rgUY_58qp3ks2kLNht-g4oqg-9on3FB4kFFhyWrOjiihP3EOTSOWklD8-nzNYMQLexE5qEYJdr4AE62epQ2OMq) | 364.421 | - 8.84 | 33.23 ± 5.19 |
| D077 - 0380 | 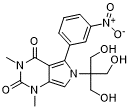 | [ChemDiv](http://www.baidu.com/link?url=lQnIdYT7ew-wpQhcPalb2rgUY_58qp3ks2kLNht-g4oqg-9on3FB4kFFhyWrOjiihP3EOTSOWklD8-nzNYMQLexE5qEYJdr4AE62epQ2OMq) | 404.379 | - 8.792 | 32.57 ± 7.72 |
| 8013 - 3348 | 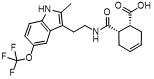 | [ChemDiv](http://www.baidu.com/link?url=lQnIdYT7ew-wpQhcPalb2rgUY_58qp3ks2kLNht-g4oqg-9on3FB4kFFhyWrOjiihP3EOTSOWklD8-nzNYMQLexE5qEYJdr4AE62epQ2OMq) | 410.392 | - 7.001 | 31.43 ± 4.24 |
| D715 - 1232 | 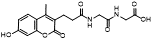 | [ChemDiv](http://www.baidu.com/link?url=lQnIdYT7ew-wpQhcPalb2rgUY_58qp3ks2kLNht-g4oqg-9on3FB4kFFhyWrOjiihP3EOTSOWklD8-nzNYMQLexE5qEYJdr4AE62epQ2OMq) | 362.338 | - 10.351 | 30.98 ± 5.98 |
| D231 - 0033 | 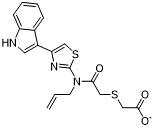 | ChemDiv | 387.471 | - 8.158 | 30.86 ± 6.95 |
| Y200 - 5859 | 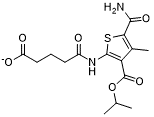 | ChemDiv | 356.393 | - 9.467 | 30.53 ± 8.55 |
| Y600 - 5089 | 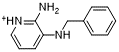 | ChemDiv | 199.255 | - 8.544 | 30.11 ± 4.35 |
| Y041 - 0706 | 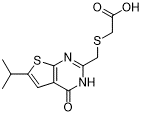 | [ChemDiv](http://www.baidu.com/link?url=lQnIdYT7ew-wpQhcPalb2rgUY_58qp3ks2kLNht-g4oqg-9on3FB4kFFhyWrOjiihP3EOTSOWklD8-nzNYMQLexE5qEYJdr4AE62epQ2OMq) | 298.374 | - 10.457 | 29.97 ± 8.09 |
| 8010 - 8861 | 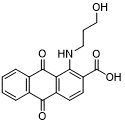 | [ChemDiv](http://www.baidu.com/link?url=lQnIdYT7ew-wpQhcPalb2rgUY_58qp3ks2kLNht-g4oqg-9on3FB4kFFhyWrOjiihP3EOTSOWklD8-nzNYMQLexE5qEYJdr4AE62epQ2OMq) | 325.32 | - 7.295 | 29.84 ± 8.96* |
| Y040 - 8306 | 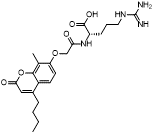 | [ChemDiv](http://www.baidu.com/link?url=lQnIdYT7ew-wpQhcPalb2rgUY_58qp3ks2kLNht-g4oqg-9on3FB4kFFhyWrOjiihP3EOTSOWklD8-nzNYMQLexE5qEYJdr4AE62epQ2OMq) | 446.502 | - 10.845 | 29.73 ± 5.69 |
| Y080 - 1087 | 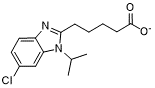 | ChemDiv | 294.78 | - 8.632 | 28.31 ± 2.12 |
| 8266 - 0242 | 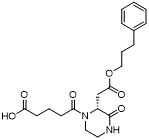 | [ChemDiv](http://www.baidu.com/link?url=lQnIdYT7ew-wpQhcPalb2rgUY_58qp3ks2kLNht-g4oqg-9on3FB4kFFhyWrOjiihP3EOTSOWklD8-nzNYMQLexE5qEYJdr4AE62epQ2OMq) | 390.435 | - 10.399 | 23.16 ± 8.44 |
| C301 - 0420 | 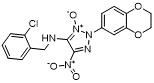 | ChemDiv | 403.781 | - 9.536 | 22.45 ± 2.05 |
| Y501 - 5661 | 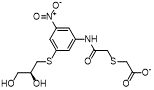 | ChemDiv | 376.399 | - 10.221 | 20.59 ± 5.49 |
| Y040 - 8596 | 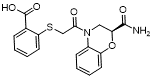 | [ChemDiv](http://www.baidu.com/link?url=lQnIdYT7ew-wpQhcPalb2rgUY_58qp3ks2kLNht-g4oqg-9on3FB4kFFhyWrOjiihP3EOTSOWklD8-nzNYMQLexE5qEYJdr4AE62epQ2OMq) | 375.386 | - 10.519 | 20.55 ± 8.09 |
| Y044 - 4612 | 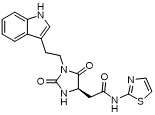 | ChemDiv | 383.424 | - 8.333 | 19.64 ± 4.57 |
| 8009 - 0443 | 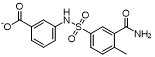 | ChemDiv | 334.346 | - 8.552 | 18.97 ± 9.60 |
| 3802 - 0568 | 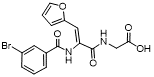 | [ChemDiv](http://www.baidu.com/link?url=lQnIdYT7ew-wpQhcPalb2rgUY_58qp3ks2kLNht-g4oqg-9on3FB4kFFhyWrOjiihP3EOTSOWklD8-nzNYMQLexE5qEYJdr4AE62epQ2OMq) | 393.193 | - 10.42 | 18.12 ± 2.19 |
| 0527 - 0129 | 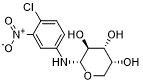 | ChemDiv | 304.686 | - 8.867 | 15.66 ± 1.23 |
| 4873 - 0243 | 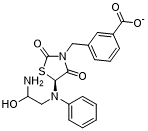 | ChemDiv | 399.42 | - 8.958 | 15.32 ± 6.09 |
| 8010 - 6264 | 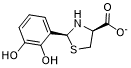 | [ChemDiv](http://www.baidu.com/link?url=lQnIdYT7ew-wpQhcPalb2rgUY_58qp3ks2kLNht-g4oqg-9on3FB4kFFhyWrOjiihP3EOTSOWklD8-nzNYMQLexE5qEYJdr4AE62epQ2OMq) | 241.261 | - 8.406 | 13.98 ± 8.31 |
| 0180 - 0412 | 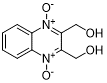 | ChemDiv | 222.2 | - 9.943 | 12.21 ± 3.81 |
| Y043 - 2512 | 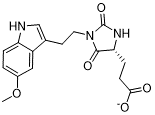 | ChemDiv | 345.354 | - 8.451 | 11.34 ± 3.58 |
| 2699 - 0153 | 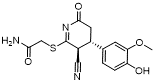 | [ChemDiv](http://www.baidu.com/link?url=lQnIdYT7ew-wpQhcPalb2rgUY_58qp3ks2kLNht-g4oqg-9on3FB4kFFhyWrOjiihP3EOTSOWklD8-nzNYMQLexE5qEYJdr4AE62epQ2OMq) | 333.361 | - 10.916 | 11.2 ± 7.19 |
| 8266 - 0231 | 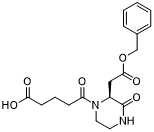 | [ChemDiv](http://www.baidu.com/link?url=lQnIdYT7ew-wpQhcPalb2rgUY_58qp3ks2kLNht-g4oqg-9on3FB4kFFhyWrOjiihP3EOTSOWklD8-nzNYMQLexE5qEYJdr4AE62epQ2OMq) | 362.382 | - 10.462 | 10.54 ± 8.44 |
| C301 - 0183 | 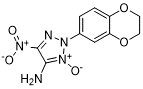 | ChemDiv | 279.212 | - 8.005 | 10.31 ± 2.48 |
| Z85881721 | 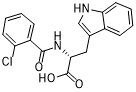 | [Enamine](http://www.baidu.com/link?url=lQnIdYT7ew-wpQhcPalb2rgUY_58qp3ks2kLNht-g4oqg-9on3FB4kFFhyWrOjiihP3EOTSOWklD8-nzNYMQLexE5qEYJdr4AE62epQ2OMq) | 342.776 | - 8.813 | 10.12 ± 8.78 |
| 0083 - 0079 | 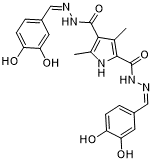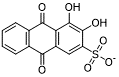 | [ChemDiv](http://www.baidu.com/link?url=lQnIdYT7ew-wpQhcPalb2rgUY_58qp3ks2kLNht-g4oqg-9on3FB4kFFhyWrOjiihP3EOTSOWklD8-nzNYMQLexE5qEYJdr4AE62epQ2OMq) | 320.273 | - 10.427 | 10.11 ± 8.54* |
| 8012 - 8947 | 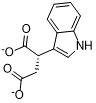 | ChemDiv | 233.223 | - 7.995 | 7.32 ± 5.97 |
| Y041 - 6417 | 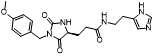 | [ChemDiv](http://www.baidu.com/link?url=lQnIdYT7ew-wpQhcPalb2rgUY_58qp3ks2kLNht-g4oqg-9on3FB4kFFhyWrOjiihP3EOTSOWklD8-nzNYMQLexE5qEYJdr4AE62epQ2OMq) | 385.422 | - 10.714 | 7.22 ± 8.78 |
| 2530 - 0128 | 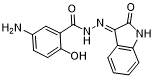 | [ChemDiv](http://www.baidu.com/link?url=lQnIdYT7ew-wpQhcPalb2rgUY_58qp3ks2kLNht-g4oqg-9on3FB4kFFhyWrOjiihP3EOTSOWklD8-nzNYMQLexE5qEYJdr4AE62epQ2OMq) | 296.285 | - 6.78 | 0* |
| 8017 - 3580 | 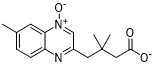 | ChemDiv | 274.319 | - 10.816 | 0 |
| 8016 - 2742 | 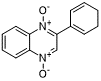 | ChemDiv | 238.245 | - 8.97 | 0 |
| 0099 - 0166 | 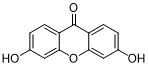 | ChemDiv | 228.204 | - 7.742 | 0 |
| 3871 - 0118 | 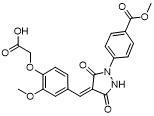 | [ChemDiv](http://www.baidu.com/link?url=lQnIdYT7ew-wpQhcPalb2rgUY_58qp3ks2kLNht-g4oqg-9on3FB4kFFhyWrOjiihP3EOTSOWklD8-nzNYMQLexE5qEYJdr4AE62epQ2OMq) | 426.382 | - 10.547 | 0 |
| 5829 - 7864 | 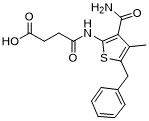 | [ChemDiv](http://www.baidu.com/link?url=lQnIdYT7ew-wpQhcPalb2rgUY_58qp3ks2kLNht-g4oqg-9on3FB4kFFhyWrOjiihP3EOTSOWklD8-nzNYMQLexE5qEYJdr4AE62epQ2OMq) | 346.4 | - 9.778 | 0 |
| C200 - 9250 | 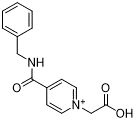 | [ChemDiv](http://www.baidu.com/link?url=lQnIdYT7ew-wpQhcPalb2rgUY_58qp3ks2kLNht-g4oqg-9on3FB4kFFhyWrOjiihP3EOTSOWklD8-nzNYMQLexE5qEYJdr4AE62epQ2OMq) | 271.2955 | - 9.958 | 0 |
| Y020 - 3003 | 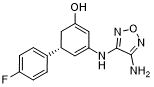 | [ChemDiv](http://www.baidu.com/link?url=lQnIdYT7ew-wpQhcPalb2rgUY_58qp3ks2kLNht-g4oqg-9on3FB4kFFhyWrOjiihP3EOTSOWklD8-nzNYMQLexE5qEYJdr4AE62epQ2OMq) | 288.281 | - 9.136 | 0 |
| Y041 - 2384 | 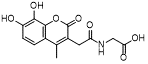 | [ChemDiv](http://www.baidu.com/link?url=lQnIdYT7ew-wpQhcPalb2rgUY_58qp3ks2kLNht-g4oqg-9on3FB4kFFhyWrOjiihP3EOTSOWklD8-nzNYMQLexE5qEYJdr4AE62epQ2OMq) | 307.259 | - 9.786 | 0 |
| Z166653136 | 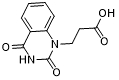 | [Enamine](http://www.baidu.com/link?url=lQnIdYT7ew-wpQhcPalb2rgUY_58qp3ks2kLNht-g4oqg-9on3FB4kFFhyWrOjiihP3EOTSOWklD8-nzNYMQLexE5qEYJdr4AE62epQ2OMq) | 234.208 | - 9.436 | 0 |
| Z812953950 | 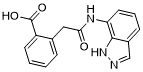 | [Enamine](http://www.baidu.com/link?url=lQnIdYT7ew-wpQhcPalb2rgUY_58qp3ks2kLNht-g4oqg-9on3FB4kFFhyWrOjiihP3EOTSOWklD8-nzNYMQLexE5qEYJdr4AE62epQ2OMq) | 295.293 | - 8.925 | 0 |
| Z242711038 | 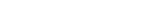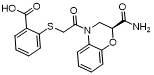 | [Enamine](http://www.baidu.com/link?url=lQnIdYT7ew-wpQhcPalb2rgUY_58qp3ks2kLNht-g4oqg-9on3FB4kFFhyWrOjiihP3EOTSOWklD8-nzNYMQLexE5qEYJdr4AE62epQ2OMq) | 372.395 | - 9.53 | 0 |
| Z951211480 | 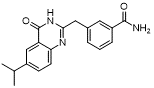 | [Enamine](http://www.baidu.com/link?url=lQnIdYT7ew-wpQhcPalb2rgUY_58qp3ks2kLNht-g4oqg-9on3FB4kFFhyWrOjiihP3EOTSOWklD8-nzNYMQLexE5qEYJdr4AE62epQ2OMq) | 321.373 | - 9.472 | 0 |
